# Phylogeny and New Classification of Hydrothermal Vent and Seep Shrimps of the Family Alvinocarididae (Decapoda)

**DOI:** 10.1371/journal.pone.0129975

**Published:** 2015-07-10

**Authors:** Alexander L. Vereshchaka, Dmitry N. Kulagin, Anastasia A. Lunina

**Affiliations:** P.P. Shirshov Institute of Oceanology of Russian Academy of Sciences, Moscow, 117997, Russia; Sars International Centre for Marine Molecular Biology, NORWAY

## Abstract

The paper addresses the phylogeny and classification of the hydrothermal vent shrimp family Alvinocarididae. Two morphological cladistic analyses were carried out, which use all 31 recognized species of Alvinocarididae as terminal taxa. As outgroups, two species were included, both representing major caridean clades: *Acanthephyra purpurea* (Acanthephyridae) and *Alpheus echiurophilus* (Alpheidae). For additional support of the clades we utilised available data on mitochondrial Cytochrome c Oxidase I gene (CO1) and 16S ribosomal markers. Both morphological and molecular methods resulted in similar tree topologies and nearly identical clades. We consider these clades as evolutionary units and thus erect two new subfamilies: Rimicaridinae (*Alvinocaridinides*, *Manuscaris*, *Opaepele*, *Shinkaicaris*, *Rimicaris*), Alvinocaridinae (*Alvinocaris*), whilst recognising Mirocaridinae (with genera *Mirocaris* and *Nautilocaris*) at subfamily level. One genus, *Keldyshicaris* could not be assigned to any subfamily and is thus left as incertae sedis. The monophyly of Alvinocardinae was supported by morphological data, but not supported by molecular data (two analyses); the monophyly of all subfamilies was supported both by morphological and molecular data. *Chorocaris* is herein synonymized with *Rimicaris*, whilst *Opaepele vavilovi* is herein transferred to a new genus *Keldyshicaris*. Morphological trends within Alvinocarididae are discussed and short biogeographical remarks are given. We provide emended diagnoses for all subfamilies and genera along with keys to all recognized species.

## Introduction

Shrimps of the family Alvinocarididae inhabit deep-sea cold-seeps and hydrothermal vent areas around the world, and have been found in the Atlantic, Pacific, and Indian Oceans [1] within the depth range of 252 to 4960 m [2–3]. Most species of the family occur at hydrothermal vents, but a few are found in cold-seep areas [3]; one species, *Alvinocaris longirostris*, has been reported from both vents and seeps [4–7]. The first record of the family was based on a few specimens from the Galapagos Rift, which were described in 1982 as *Alvinocaris lusca* by Austin Williams and Fenner Chace [8]. Later the first author described a further two species of a new genus, *Rimicaris* from the hydrothermal vent field TAG [9]. One of these species was subsequently transferred to a new genus *Chorocaris* in 1990 [10]. In the middle of the 1990s, Russian and American scientists described two further genera *Opaepele* [11] and *Mirocaris* [12]; whilst more recently the genera *Nautilocaris*, *Shinkaicaris*, and *Alvinocaridinides* were described by Japanese and French researchers [2], [13], [14].

Due to drastic metamorphosis in ontogeny, the history of the family systematics has not been smooth and some taxa were later synonimised. For example, the genus *Iorania* [15] and the species *Rimicaris aurantiaca* [16] are now considered to be juveniles of *Rimicaris exoculata*. There was no consensus on the status of the new family Mirocarididae established for a single genus *Mirocaris* [12]; phylogenetics showed a significant distance between this group and the rest of Alvinocarididae [17–18], although taxonomists kept *Mirocaris* as a genus within Alvinocarididae [19]. Status for *Opaepele vavilovi* also remains unseratin [20].

Three new species and a new genus *Manuscaris* have recently been described from hydrothermal vents in the Pacific Ocean [21]. In this comprehensive study, partial sequences of mitochondrial COI were used, resulting in a minor change in the classification of Alvinocarididae, the transfer of *Opaepele susannae* into *Chorocaris* [21].

At present, 9 genera and 31 species are known within the family and a comprehensive phylogenetic analysis is needed to disentangle existing problems and to elucidate the status of all genera.

This task is eased by the presence of a significant amount of information on partial sequences of mitochondrial COI gene in GenBank, unusually rich for decapods. Much of this data was used in a previous comprehensive study [17], which confirmed three distinct clades consistent with morphology at that time: (1) *Rimicaris/Chorocaris/Opaepele*, (2) *Alvinocaris*, and (3) *Mirocaris*. Evolutionary relationships of vent-endemic shrimp species were shown to correlate neither with their current biogeographic distribution nor with the history of sea-floor spreading. Later studies have incorporated further molecular data for several recently described species and enhanced information for the species studied in [3], [17], [18], [21–26]. However, no attempt to carry out a complete phylogenetic analysis of the whole family Alvinocarididae has been carried out to date.

Combining both morphological and molecular evidence should shed light on the complex relationships in Alvinocarididae.

In this paper we summarize original and literature data about the composition, morphology, and genetic diversity of the family Alvinocarididae. Further, we (1) find and describe morphological characters, (2) perform cladistic morphological analyses, (3) analyze molecular data, (4) combine and compare morphological and molecular results, (5) discuss supported taxa, and (6) provide a new classification, emended diagnoses, and identification keys for all subfamilies, genera, and species.

## Material and Methods

### Material for morphological analysis

Material was collected along the Mid-Atlantic Ridge during six cruises of R/V “Akademik Mstislav Keldysh” with the use of two deep-sea manned submersibles "Mir–1" and "Mir–2" (34th cruise, August-October 1994, 39th cruise, August-October 1996, 41st cruise, August-December 1998, 47th cruise, June-July 2002, 49th cruise August 2003, 50th cruise, August 2005). Seven vent fields were investigated during 1994–2005, including Menez Gwen (37.8417 N 31.525 W), Lucky Strike (37.2933 N 32.2733 W), Rainbow (36.23 N 33.902 W), Broken Spur (29.17 N 43.1717 W), TAG (26.1367 N 44.8267 W), Snake Pit (23.3683 N 44.95 W) and Logatchev (14.752 N 44.9785 W). No specific permission was required for field studies in any of these locations. The field studies did not involve endangered or protected species.

Shrimps were collected using baited traps and suction samplers. Immediately after retrieval all specimens were sorted, measured, and preserved in 80% alcohol. Measurements follow established methods for shrimp morphological description [27]. Shrimp morphology and its temporal/spatial variations were thoroughly investigated for this material on the basis of 5861 individuals [28], [29]. A detailed description of this material and discussion of the various species may be found in [20], [29], [30].

Analysis of the morphology of all species within the family was made with the use the above original data and all other available literature data (Table 1).

**Table 1:** List of all valid species of the family Alvinocarididae, with remarks on their former and current status.

Genus			Species	Description, author and year	Type locality, depth
Before (Komai, Tsuchida, 2015)	After (Komai, Tsuchida, 2015)	Here			
*Alvinocari-dinides*	*Alvinocari-dinides*	*Alvinocari-dinides*	*formosa*	Komai, Chan, 2010	Gueishandao, Yilan County, Taiwan, 24°51.231'N 0121°59.204'E, 252–275 m
*Alvinocaris*	*Alvinocaris*	*Alvinocaris*	*alexander*	Ahyong, 2009	Rumble V Seamount, 36°08.27–07.96'S 78°11.74–11.70'E, 485–415 m
*Alvinocaris*	*Alvinocaris*	*Alvinocaris*	*brevitel-sonis*	Kikuchi, Hashimoto, 2000	”Depression C” of the Minami-Ensei Knoll, 28°23.35'N 127°38.38'E, 705 m
*Alvinocaris*	*Alvinocaris*	*Alvinocaris*	*chelys*	Komai, Chan, 2010	Gueishandao, Yilan County, Taiwan, 24°49.682'N 122°0.254'E, 300–276 m
*Alvinocaris*	*Alvinocaris*	*Alvinocaris*	*dissimilis*	Komai, Segonzac, 2005	Depression C, Minami-Ensei Knoll, 28°23.35'N 127°38.38'E, 705 m
*Alvinocaris*	*Alvinocaris*	*Alvinocaris*	*komaii*	Zelnio, Hourdez, 2009	Kilo Moana, Eastern Lau Spreading Center, Lau Basin, southwest Pacific,; 20°9'S 76°12'E, 2620 m
*Alvinocaris*	*Alvinocaris*	*Alvinocaris*	*longi-rostris*	Kikuchi, Ohta, 1995	Iheya Ridge, Clam Site, Okinawa Trough, 27°32.70'N 126°58.20'E, 1360 m
*Alvinocaris*	*Alvinocaris*	*Alvinocaris*	*lusca*	Williams, Chace, 1982	Galapagos Rift Rose Garden area, 0°48.25'N 86°13.48'W, maximum of 2450 m
*Alvinocaris*	*Alvinocaris*	*Alvinocaris*	*markensis*	Williams, 1988	Mid-Atlantic Rift Valley about 70 km south of Kane Fracture Zone, 23°22.09'N 44°57.12'W, 3437 m
*Alvinocaris*	*Alvinocaris*	*Alvinocaris*	*methano-phila*	Komai, Shank, Van Dover, 2005	ODP site 996, Blake Ridge Diapir, 32°29.623'N 76°11.467'W, 2155 m
*Alvinocaris*	*Alvinocaris*	*Alvinocaris*	*muricola*	Williams, 1988	West Florida Escarpment, 26°01'N 84°54.61'W, 3277 m
*Alvinocaris*	*Alvinocaris*	*Alvinocaris*	*niwa*	Webber, 2004	Rumble V, 36°8.63–8.57'S 178°11.77–11.50'E, 877–655 m
*Alvinocaris*	*Alvinocaris*	*Alvinocaris*	*stactophila*	Williams, 1988	north central Gulf of Mexico about 129 km S of Louisiana, 27°46.94'N 91°30.34'W, 534 m
*Alvinocaris*	*Alvinocaris*	*Alvinocaris*	*solitaire*	Yahagi, Watanabe, Kojima, Beedessee, Komai, 2014	Central Indian Ridge, Solitaire hydrothermal vent field,19°33.413’S, 65°50.888’E, 2606 m
*Alvinocaris*	*Alvinocaris*	*Alvinocaris*	*williamsi*	Shank, Martin, 2003	Menez Gwen hydrothermal field, North Atlantic Ocean, 37°50.5'N 31°31.3'W, 850 m
*Chorocaris*	*Chorocaris*	*Rimicaris*	*chacei*	(Williams, Rona, 1986)	TAG Hydrothermal Field, Mid-Atlantic Ridge, 26°08.3'N 44°49.6'W, 3620–3650 m
*Chorocaris*	*Chorocaris*	*Rimicaris*	*paulexa*	Martin, Shank, 2005	Homer Vent (347OC black smoker), 17°37.220'S 113°15.123'W, 2595 m, southern East Pacific Rise
-	*Chorocaris*	*Rimicaris*	*parva*	Komai, Tsuchida, 2015	Manus Basin, South Su, Wave Mercury 2007 (Luk Luk) Campaign, 03°08.09’S, 152°10.5’E, 1310 m
*Chorocaris*	*Chorocaris*	*Rimicaris*	*van-doverae*	Martin, Hessler, 1990	Alice springs vent field, Mariana Back-Arc Basin, 18°12.599'N 144°42.431'E, 3640 m
-	*Chorocaris*	*Rimicaris*	*variabilis*	Komai, Tsuchida, 2015	Manus Basin, South Su, Wave Mercury 2007 (Luk Luk) Campaign, 03°08.09’S, 152°10.5’E, 1310 m
*Mirocaris*	*Mirocaris*	*Mirocaris*	*fortunata*	(Martin, Christiansen, 1995)	Vent site 3, Lucky Strike hydrothermal vent, Azores, 37°17.6'N 32°16.5'W, 1624 m
*Mirocaris*	*Mirocaris*	*Mirocaris*	*indica*	Komai, Martin, Zala, Tsuchida, Hashimoto, 2006	Central Indian Ridge, Kairei Field, 25°19.2'S 70°02.4'E, 2422 m
-	*Manuscaris*	*Manuscaris*	*acumi-natus*	Komai, Tsuchida, 2015	Manus Basin, South Su, Wave Mercury 2007 Campaign, 03°08.09’S, 152°10.5’E, 1310 m
*Nautilocaris*	*Nautilocaris*	*Nautilocaris*	*saint-laurentae*	Komai, Segonzac, 2004	North Fiji Basin, White Lady site, 16°59.50'S 173°55.47'E, 2000 m
*Opaepele*	*Opaepele*	*Opaepele*	*loihi*	Williams, Dobbs, 1995	Loihi Seamount, Hawaii, 18°55'N 155°16'W, 980 m
*Opaepele*	*Chorocaris*	*Rimicaris*	*susannae*	Komai, Giere, Segonzac, 2007	Lilliput, southern Mid-Atlantic Ridge, 09°32.845'S 13°12.546'W, 1500 m, mussel field with diffuse vent fluids
*Opaepele*	*Opaepele*	*Keldyshi-caris*	*vavilovi*	Lunina, Vereshchaka, 2010	Mid-Atlantic Ridge, Broken Spur vent site, stn 4797
*Rimicaris*	*Rimicaris*	*Rimicaris*	*exoculata*	Williams, Rona, 1986	TAG Hydrothermal Field, Mid-Atlantic Ridge, 26°08.3'N 44°49.6'W, 3620–3650 m
*Rimicaris*	*Rimicaris*	*Rimicaris*	*hybisae*	Nye, Copley, Plouviez, 2011	Mid-Cayman Spreading Centre, Caribbean, Von Damm vent field, 18822.605′N81847.875′W; 2300 m
*Rimicaris*	*Rimicaris*	*Rimicaris*	*kairei*	Watabe, Hashimoto, 2002	The Central Indian ridge, Indian Ocean, the Kairei Field, 25°19.16'S 70°02.40'E, 2454 m
*Shinkaicaris*	*Shinkaicaris*	*Shinkaicaris*	*leurokolos*	(Kikuchi, Hashimoto, 2000)	”Depression C” of the Minami- Ensei Knoll, 28°23.35'N 127°38.38'E, 705 m

### Terminal taxa, outgroups, and characters used for morphological analysis

All thirty-one recognized species of Alvinocarididae were included as terminals. Outgroup selection was made on the basis of a comprehensive molecular study [31], which revealed two major clades of Caridea: (1) Alpheidae, Hippolytidae, Crangonidae, Glyphocrangonidae, Barbouriidae, Pandalidae, Hymenoceridae, Gnathophyllidae, and Palaemonidae and (2) Rhynchocinetidae, Oplophoridae, Nematocarcinidae, Alvinocarididae, Campylonotidae, Pasiphaeidae and Eugonatonotidae. The first outgroup species, *Acanthephyra purpurea* A. Milne-Edwards, 1881 [32], represents the first clade: Wong et al. [33] have shown that family Acanthephyridae is sister to Oplophoridae and advocate combining both families as Oplophoridae. We chose *A*. *purpurea* partly because this species is present in GenBank and could also be used as the outgroup in the molecular analysis. The second outgroup species, *Alpheus echiurophilus* Anker, Komai and Marin 2015 [34], belongs to Alpheidae and represents the second major clade of Caridea. Both species are ecologically very different (pelagic and burrowing) as well as morphologically and a comparison of cladograms is thus instructive.

**Fig 1:**
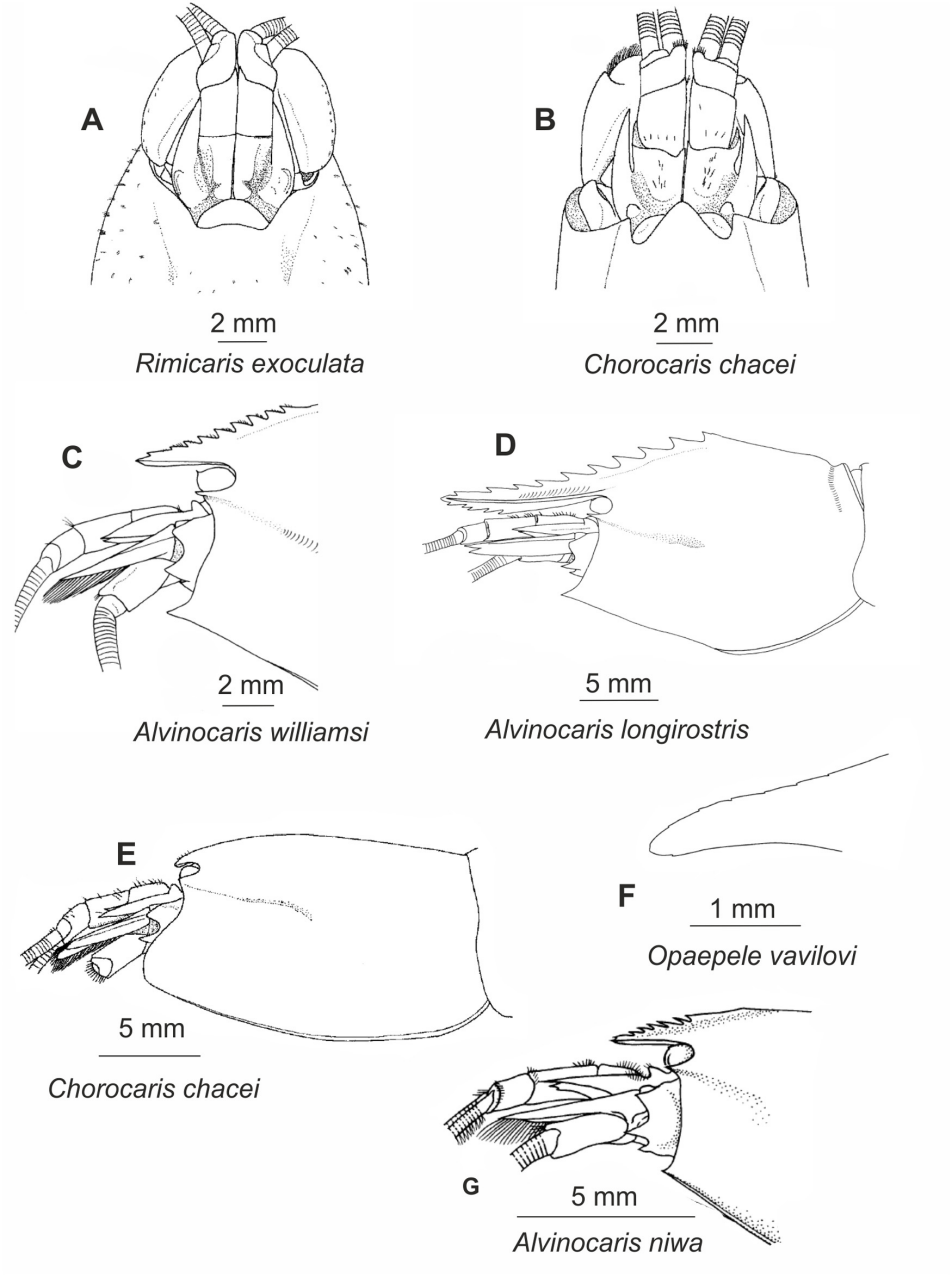
Morphological characters used for phylogenetic analysis. Anterior part of body. (A)-(E) after [13], (F) after [20], (G) after [70].

**Fig 2:**
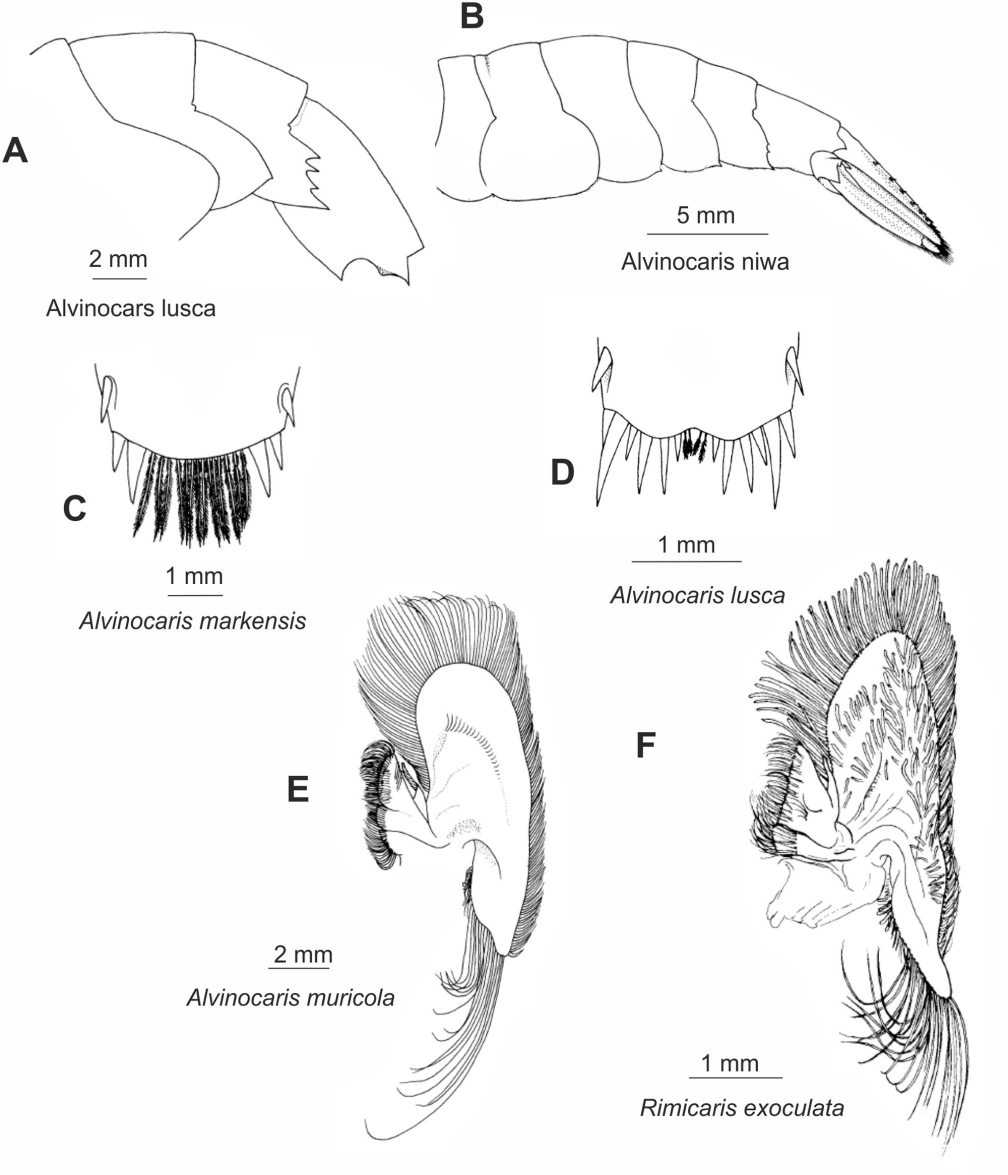
Morphological characters used for phylogenetic analysis. Posterior part of body and maxilla. (A), (C)-(E) after [13], (B) after [70], (F) after [9].

**Fig 3:**
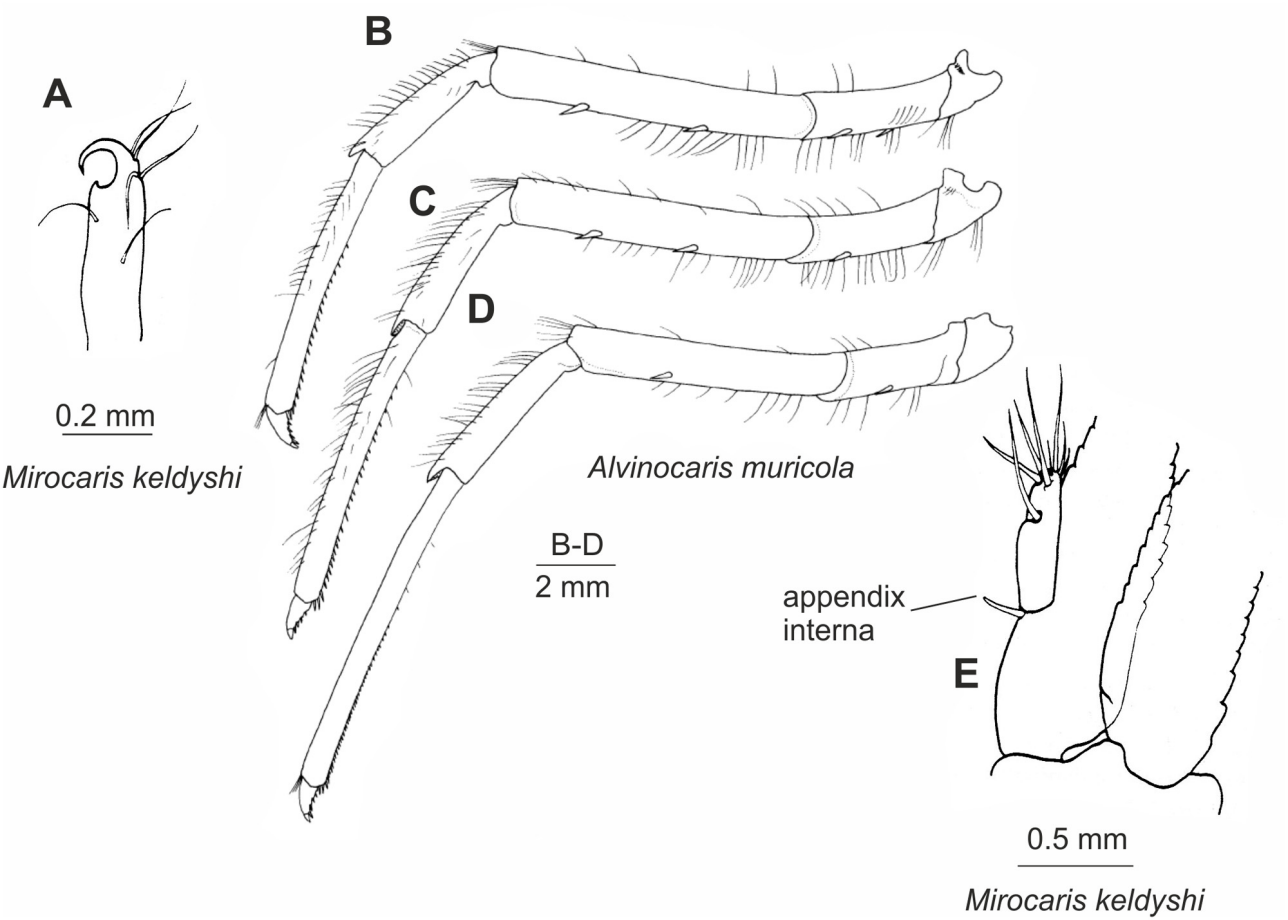
Morphological characters used for phylogenetic analysis. Thoracic and pleonic appendages. (A), (E) after [12], (B)-(D) after [13].

Sixty-three morphological characters (ten multistate) were used in the analysis, and are listed in Table 2, along with character states, brief descriptions, and references to figures (see also Figs 1–3). The data matrix is presented in Table 3.

**Table 2:** List of morphological characters and their states.

No	Character	Character state	State No	Figure
**CARAPACE**				
0	Rostrum	absent	0	1A
		present	1	1B
1	Rostrum	not reaching end of 1^st^ antennular segment	0	1B
		reaching end of 1^st^ antennular segment	1	1C
		overreaching end of 2nd antennular segment	2	1D
2	Rostrum,	tip acute	0	1C
		tip obtuse	1	1B
3	Rostrum	laterally compressed	0	1C
		not laterally compressed	1	1B
4	Rostrum	not dorsoventrally compressed	0	1C
		dorsoventrally compressed	1	1B
5	Rostrum	dorsally carinate	0	1C
		not dorsally carinate	1	1B
6	Rostrum	dorsally smooth	0	1E
		dorsally notched	1	1F
		dorsally toothed	2	1C,D
7	Rostrum, minimal number of dorsal teeth or notches	0	0	
		5–10	1	
		11–15	2	
		16 or more	3	
8	Rostrum, maximal number of dorsal teeth or notches	0	0	
		4–10	1	
		11–15	2	
		16 or more	3	
9	Rostrum	ventrally carinate	0	1C
		not ventrally carinate	1	1B
10	Rostrum	ventrally smooth	0	1E
		ventrally notched	1	1F
		ventrally toothed	2	1D
11	Rostrum, minimal number of ventral teeth or notches	0	0	
		1–2	1	
		3–6	2	
12	Rostrum, maximal number of ventral teeth or notches	0	0	
		1–2	1	
		6–11	2	
13	Rostrum, minimal number of teeth or notches on carapace	0	0	
		1–5	1	
		6–10	2	
14	Rostrum, maximal number of teeth or notches on carapace	0	0	
		1–5	1	
		6–10	2	
15	Carapace, postrostral dorsal carina extending beyond the midlength	absent	0	
		present	1	
16	Carapace, antennal angle	blunt	0	
		acute	1	
17	Carapace, acute pterygostomial tooth	absent	0	
		present	1	
18	Dorsal organ under carapace	absent or inconspicuous	0	
		conspicuous	1	
19	Dorsal organ under carapace	restricted to postorbital region	0	
		extended beyond the postorbital region	1	
20	Dorsal organ	nearly entire	0	
		four-lobed, without pores	1	
		four-lobed, with a pore	2	
**ABDOMEN AND TELSON**				
21	Third abdominal segment, posterior margin of pleura	smooth	0	2A
		serrated	1	2B
22	Telson, long linear row of movable dorsolateral spines (≥5 in row)	absent	0	
		present	1	
23	Telson, long sinuous row of movable dorsolateral spines (≥5 in row)	absent	0	
		present	1	
24	Telson, number of strong spines on posterior margin	2–4	0	2C
		6 or more	1	2D
25	Telson, posterior margin	convex	0	2C
		concave	1	2D
26	Telson, posterior concave margin	with shallow incision	0	2D
		nearly bilobed	1	
**THORACIC APPENDAGES**				
27	Eyestalks	not fused partly	0	
		fused partly, mould seam present	1	
28	Eyestalks	not fused entirely	0	
		fused entirely, without mould seam	1	
29	Eyes, anterior margin	entire	0	
		with conspicuous tubercle	1	1C
30	Antenna II	not operculiform	0	
		operculiform	1	
31	Maxilla II, plumose bacteriophorous setae on scaphognathite	absent	0	2E
		present	1	2F
32	Maxilliped III, epipod	subtriangular	0	
		strap-like	1	3A
33	Maxilliped III, epipod	not terminated in hook	0	
		terminated in hook	1	3A
34	Maxilliped III, merus	unarmed	0	
		with 1–2 distal spines	1	
35	Pereopod I, epipod	absent or rudimentary	0	
		strap-like	1	3A
36	Pereopod I, epipod	not terminated in hook	0	
		terminated in hook	1	3A
37	Pereopod I, grooming apparatus	absent or inconspicuous	0	
		conspicuous	1	
38	Pereopod II, epipod	absent or rudimentary	0	
		strap-like	1	3A
39	Pereopod II, epipod	not terminated in hook	0	
		terminated in hook	1	3A
40	Pereopod II, movable spines on ischium	absent	0	
		present	1	
41	Pereopod III, epipod	absent or rudimentary	0	
		strap-like	1	3A
42	Pereopod III, epipod	not terminated in hook	0	
		terminated in hook	1	3A
43	Pereopod III, strong movable spines on ischium	absent	0	
		present	1	3B
44	Pereopod III, proximal strong movable spines on merus	absent	0	
		present	1	3B
45	Pereopod III, distal movable spines on merus	absent	0	
		present	1	3B
46	Pereopod III, dactyl	single row of accessory spinules absent	0	
		single row of accessory spinules present	1	
47	Pereopod III, dactyl	two or more rows of accessory spinules absent	0	
		two or more rows of accessory spinules present	1	
48	Pereopod IV, epipod	absent or rudimentary	0	
		strap-like	1	3A
49	Pereopod IV, epipod	not terminated in hook	0	
		terminated in hook	1	3A
50	Pereopod IV, strong movable spines on ischium	absent	0	
		present	1	3C
51	Pereopod IV, proximal strong movable spines on merus	absent	0	
		present	1	3C
52	Pereopod IV, distal movable spines on merus	absent	0	
		present	1	3C
53	Pereopod IV, dactyl	single row of accessory spinules absent	0	
		single row of accessory spinules present	1	
54	Pereopod IV, dactyl	two or more rows of accessory spinules absent	0	
		two or more rows of accessory spinules present	1	
55	Pereopod V, strong movable spines on ischium	absent	0	
		present	1	3D
56	Pereopod V, dactyl	single row of accessory spinules absent	0	
		single row of accessory spinules present	1	
57	Pereopod V, dactyl	two or more rows of accessory spinules absent	0	
**ABDOMINAL APPENDAGES**				
58	Pleopod II, appendix interna	developed	0	-
		much reduced	1	3E
59	Pleopod III, appendix interna	developed	0	-
		much reduced	1	3E
60	Pleopod IV, appendix interna	developed	0	-
		much reduced	1	3E
61	Uropodal exopod, a single movable spine mesial to posterolateral tooth	absent	0	-
		present	1	-
62	Uropodal exopod, two movable spines mesial to posterolateral tooth	absent	0	-
		present	1	-

**Table 3:** The data matrix of morphological characters of Alvinocarididae.

Species	States of characters
*Acanthephyra purpurea*	120101 2 111 2 22000100–0000 0–0000010110110110 1 111010 1 111011000000
*Alpheus echiurophilus*	100111 0 001 0 00000000–0000 0–0010011011011111 1 000000 1 000010000010
*Alvinocaris alexander*	110000 2 130 2 11121110–0100 0–1010000100100100[01]111000 1 111001000010
*Alvinocaris brevitelsonis*	120000 2 220 2 12111110–0100 0–1010000100100100 1 111000 1 111001000010
*Alvinocaris chelis*	110000 2 230 2 01121110–0100 0–1010000100100100 0 111000 0 111001000010
*Alvinocaris dissimilis*	120000 2 230 2 11121110–0100 0–1010000100100100 1 111000 1 1110?1000010
*Alvinocaris komaii*	120000 2 120 2 12121110–0101 1 11000000100100100 1 110100 1 110110100010
*Alvinocaris longirostris*	120000 2 120 2 22121110–0100 0–1010000100100100 1 111000 1 111001000010
*Alvinocaris lusca*	110000 2 120 2 12111110–0101 0–1010000100100100 1 111000 1 111011000010
*Alvinocaris markensis*	110000 2 230 2 22121110–1101 1 01010000100100100 1 111000 1 111011000010
*Alvinocaris methanophila*	110000 2 230 2 22121110–0100 0–1010000100100100 1 111000 1 111001000010
*Alvinocaris muricola*	120000 2 130 2 22121110–0100 0–1010000100100100 1 111000 1 111011000010
*Alvinocaris niwa*	110000 2 110[02]11001110–1100[01]01010000100100100 1 111000 1 1110?1000010
*Alvinocaris williamsi*	110000 2 120 0 00121110–0101 0–1010000100100100 1 111000 1 1110?1000010
*Alvinocaris solitaire*	120000 2 330 2 11221110–1101 1 01010000100100100 1 111000 1 111011000010
*Alvinocaris stactophila*	110000 2 230 2 11221110–0101 0–1010000100100100 1 111000 1 1110?1000010
*Alvinocaridinides formosa*	100111 2 231 0 00000111–0010 0–0100000100100000[01]000100[01]000100100010
*Rimicaris chacei*	101111 0 001 0 00000001100010 0–0100000100100000 0 000100 0 000100100001
*Rimicaris paulexa*	101111 0 001 0 00000011100010 0–0100000100100000 0 000100 0 000100100001
*Rimicaris vandoverae*	101111 0 001 0 00000011100010 0–0100000100100000 0 000100 0 000100100001
*Rimicaris parva*	101111 0 001 0 00000101100010 0–0100000100100000 0 000100 0 000100100001
*Rimicaris variabilis*	101111 0 001 0 00000111100010 0–0100000100100000 0 000100 0 000100100001
*Rimicaris susannae*	101111 0 001 0 00000101100010 0–0100000100100000 0 000100[01]000100100001
*Rimicaris exoculata*	0–1111 0 001 0 00000001110010 0–0101100000000000 0 000100 0 000100100001
*Rimicaris kairei*	0–1111 0 001 0 00000001110010 0–0101100000000000 0 000100 0 000100100001
*Rimicaris hybisae*	101111 0 001 0 00000001120010 0–0100100100100000 0 000100 0 000100100001
*Rimicaris loihi*	101111[01]011 1 01000111001010 0–0100000100100000 0 000100 0 000100100010
*Shinkaicaris leurokolos*	110100 2 111 0 00110111000010 0–0100000100100000 0 000100 0 000100100010
*Manuscaris acuminata*	110100 2 221 0 00110111001100 0–0100000100100100 1 000100 0 000100100010
*Mirocaris fortunata*	101111 0 001 0 00000111000011 0–1000011111111111 1 001011[01]001001011110
*Mirocaris indica*	101111 0 001 0 00000111000011 0–1000011111111111 1 001011[01]001001011110
*Nautilocaris saintlaurentae*	110111 2 011 0 01000111001011 0–1000011111111111 1 001011 1 001001011110
*Keldyshicaris vavilovi*	101111 1 111 1 01000111001101 0–1010000100100100 1 001000 1 001011000010

Missing data indicated by question marks (?); inapplicable data by hyphens (-); polymorphism indicated by brackets [01]

### Analytical method for cladistic analysis

Data were analyzed using a combination of programs by maximum parsimony: Winclada/Nona, TNT, and Mesquite [35–37].

All characters were unordered (non-additive) and equally weighted, missing data were scored as unknown. Characters were unordered, so the score given for each state (i.e., 0, l, 2) implies nothing about order in a transformation series [38]. Trees were generated in TNT under the implicit enumeration. Relative stability of clades was assessed by standard bootstrapping (sample with replacement) with 10000 pseudoreplicates and by Bremer support (algorithm TBR, saving up to 10000 trees up to 3 steps longer).

### Molecular data

Both Mitochondrial Cytochrome c Oxidase I (CO1) and 16S ribosomal markers were selected for phylogenetic analyses, as only these markers have been sequenced for a representative number of alvinocaridid species, with CO1 sequences for 20 (out of 31) species available (Table 4). For the present phylogenetic analyses, we used all publicly available 271 CO1 sequences for individuals identified to species-level. Partial 16S sequences are only available for 10 alvinocaridid species (Table 4). For the present phylogenetic analyses, we used all 29 sequences available in the GeneBank.

**Table 4:** GenBank accession numbers for COI and 16S sequences of species used for phylogenetic analyses.

Species	NCBI GenBank Accession number, Locality	Reference
*Alvinocaris dissimilis*	AB779491–AB779494	[72],[73]
*Alvinocaris komaii*	EU031816 Eastern Lau Spreading Center, southwest Pacific	[18]
*Alvinocaris longirostris*	AB222050, AB222051 Hatoma Knoll, Okinawa Trough	[73], [22]
	NC020313, JQ035659 Hatoma Knoll, Okinawa Trough	[25]
	AB821296 Hatoma Knoll, Okinawa Trough	[72]
	GQ131897	[74]
*Alvinocaris lusca*	AF125404-AF125407 Galapagos hydrothermal vent field; 9°50’N hydrothermal vent field	[17]
*Alvinocaris markensis*	KC840879-KC840886, KC840893 Logatchev, Mid-Atlantic Ridge (MAR)	[44]
	AF125408, AF125409 Snake Pit, MAR	[17]
*Alvinocaris muricola* (including *Alvinocaris aff*. *muricola)*	KC840887-KC840892, KC840894-KC840927 Gulf of Mexico, GC852 site; Regab, West Africa	[45]
	EU031814, EU031815 Gulf of Mexico	[75]
	EU868627, EU868628	[76]
*Alvinocaris solitaire*	LC007114 Solitaire hydrothermal vent field, Central Indian Ridge	[69]
*Alvinocaris stactophila*	AF125410, AF125411 Louisiana Slope, Gulf of Mexico	[17]
*Alvinocaris chelys*	NC 018778, JX184903 vent field off Gueishandao (or Kueishan Island), Yilan County, northeastern Taiwan	[77]
*Alvinocaris methanophila*	AY163260 the Blake Ridge Diapir, Caribbean	[45],[69]
*Chorocaris chacei*	AF125395-AF125397, AF125414, AF125415 Snake Pit, TAG, MAR	[17]
	KC840928-KC840940 Logatchev, Lucky Strike, MAR	[45]
	AM076957, Lucky Strike, MAR	[78]
	AM087920- AM087922	[79]
*Chorocaris parva*	AB772278, AB772282 PACMANUS, Manus Basin, South West Pacific	[21]
*Chorocaris vandoverae*	AF125417, AF125418 Alice Springs, Mariana Back-Arc Basin	[17]
*Chorocaris variabilis*	AB772279-AB772281, AB7722 PACMANUS, Manus Basin, South West Pacific	[21]
*Mirocaris fortunate* (including *Mirocaris keldyshi*)	AF125424-AF125429, AF125430-AF125433 Lucky Strike, Broken Spur, Menez Gwen, TAG, Logatchev, MAR	[17]
	FJ769225, FJ769226 Menez Gwen, MAR	[23]
	AM076959, Lucky Strike, MAR	[78]
	AM087916- AM087919	[79]
*Nautilocaris saintlaurentae*	NC021971, KF226726 vent the Tofua Arc (Tonga)	[80]
*Opaepele loihi*	DQ328819-DQ328838 Marianca Arc	[81]
	AF125436, AF125437 Loihi Seamount	[17]
	NC020311, JQ035657 Nikko Seamount, Philippine Sea Plate	[25]
*Rimicaris exoculata*	AF125398-AF125403, AF125419, AF125420, AF125440 TAG, Lucky Strike, Broken Spur, Rainbow	[17]
	AF044057 MAR	[82]
	FN392996-FN393005 Rainbow, TAG, Logatchev, South MAR	[24]
	HM125910-HM125956 Rainbow, TAG, Logatchev, Ashadze, South MAR	[83]
	AF035459, TAG, South MAR	[82]
	AM087923-AM087925	[79]
	AM076958, Lucky Strike, MAR	[78]
*Rimicaris kairei*	AB813087-AB813108 Dodo, Solitaire, Edmond and Kairei fields	[84]
	NC020310, JQ035656 Kairei Field (Rodriguez Triple Junction)	[25]
*Rimicaris hybisae*	JN850606, JN850607 Beebe and Von Damm vent fields (Caribbean)	[3]
	KJ566968-KJ5678003 Beebe and Von Damm vent fields (Caribbean)	[85]
*Acanthephyra purpurea*	GU183787, GU183788	[86]
	KP075887, KP075899	[87]

### Analysis of molecular data

Multiple alignments were made with the use of the Clustal W algorithm [39]. Six CO1 sequences were discarded after alignment, as they represented non-barcoding parts of the CO1 gene or were too short. The remaining 265 aligned sequences were trimmed according to the shortest sequences (Accession Numbers: KC840928-KC840940, HM125910-HM125956) with a total length of 471 bp. Amino acid sequences received from the nucleotide sequences had no stop codons within the open reading frame using the invertebrate mitochondrial code. All 16S sequences after alignment were trimmed according to the shortest sequences (Accession Numbers: AM087916- AM087925) with a total length of 286 bp.

Phylogenetic analysis was performed using both Maximum Likelihood (ML) and Bayesian analyses. To root the resultant trees, *Acanthephyra purpurea* Awas used (also see section 2.2). The best-fit model selected using jModelTest 2.1.7 [40] was the Tamura-Nei model with a gamma distribution and invariable sites (TrN+G+I) for CO1 and the Hasegawa-Kishino-Yano model with a gamma distribution (HKY+G) for 16S data set. These models were used to generate ML gene trees in MEGA 5. Support for branches was assessed using bootstrap analyses with 1,000 replicates [41]. Bayesian phylogenetic analysis was made with the use of MrBayes v3.2.1 [42]. A general time-reversible model (GTR) of sequence evolution with a gamma distribution and invariable sites for CO1 data set was chosen as it represents the closest approximation of the Tamura—Nei model in MrBayes. HKY+G model was used for 16S data set. The Markov Chain Monte Carlo (MCMC) analysis was further used with the following settings: (1) for CO1–18 million generations, trees sampled every 5000 generation, and the first 900 trees discarded; (2) for 16S–1.5 million generations, trees sampled every 1000 generation, and the first 375 trees discarded. The average standard deviation of split frequencies between two runs of MCMC was less than 1% for each analysis, thus indicating convergence.

### Estimation of clade robustness

For morphological analyses, we considered the clades robust if they received simultaneous Bremer support ≥3 after both analyses. For molecular analyses, we considered the clades robust if they received Bayesian posterior probability value 75%.

The electronic edition of this article conforms to the requirements of the amended International Code of Zoological Nomenclature, and hence the new names contained herein are available under that Code from the electronic edition of this article. This published work and the nomenclatural acts it contains have been registered in ZooBank, the online registration system for the ICZN. The ZooBank LSIDs (Life Science Identifiers) can be resolved and the associated information viewed through any standard web browser by appending the LSID to the prefix "http://zoobank.org/". The LSID for this publication is: urn:lsid:zoobank.org:pub: XXXXXXX. The electronic edition of this work was published in a journal with an ISSN, and has been archived and is available from the following digital repositories: PubMed Central, LOCKSS.

## Results

### Morphological clades

**Fig 4:**
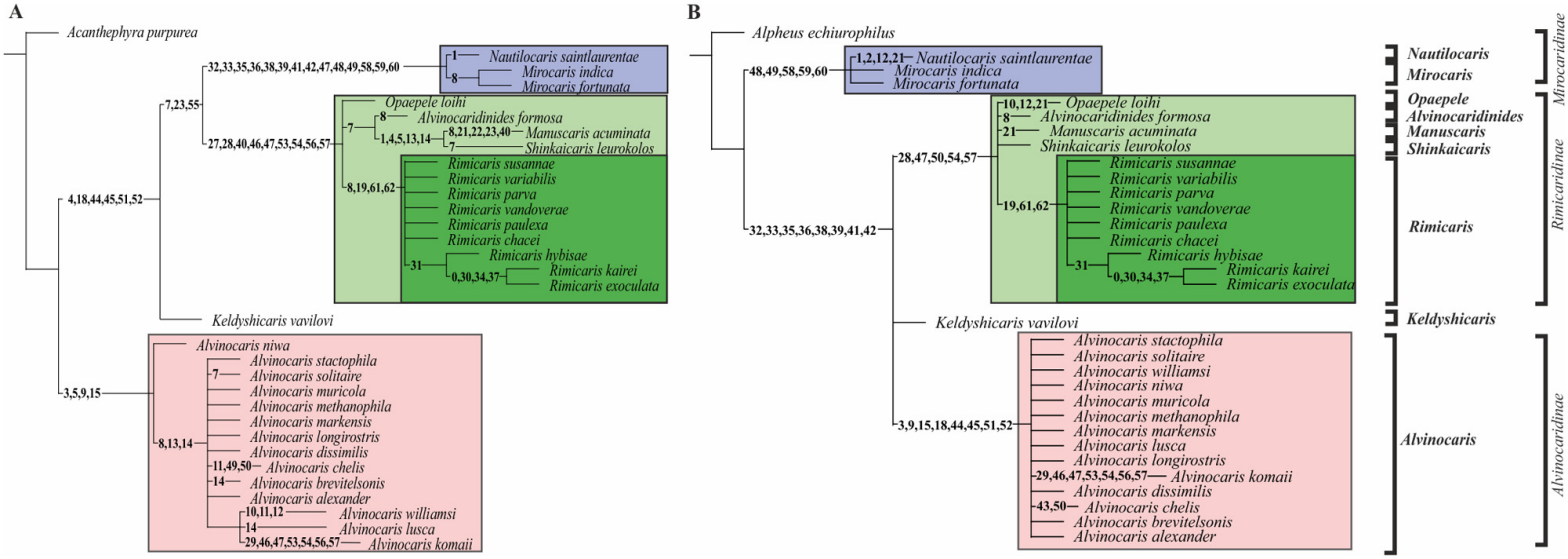
Strict consensus trees of Alvinocarididae and synapomorphies (numbers in circles). A, analysis 1 with *Acanthephyra purpurea* as the outgroup. B, analysis 2 with *Alpheus echiurophilus* as the outgroup. Supported clades are marked by different colors: Mirocaridinae (blue), Alvinocaridinae (pink), Rimicaridinae (light green), *Rimicaris* (green).

**Fig 5:**
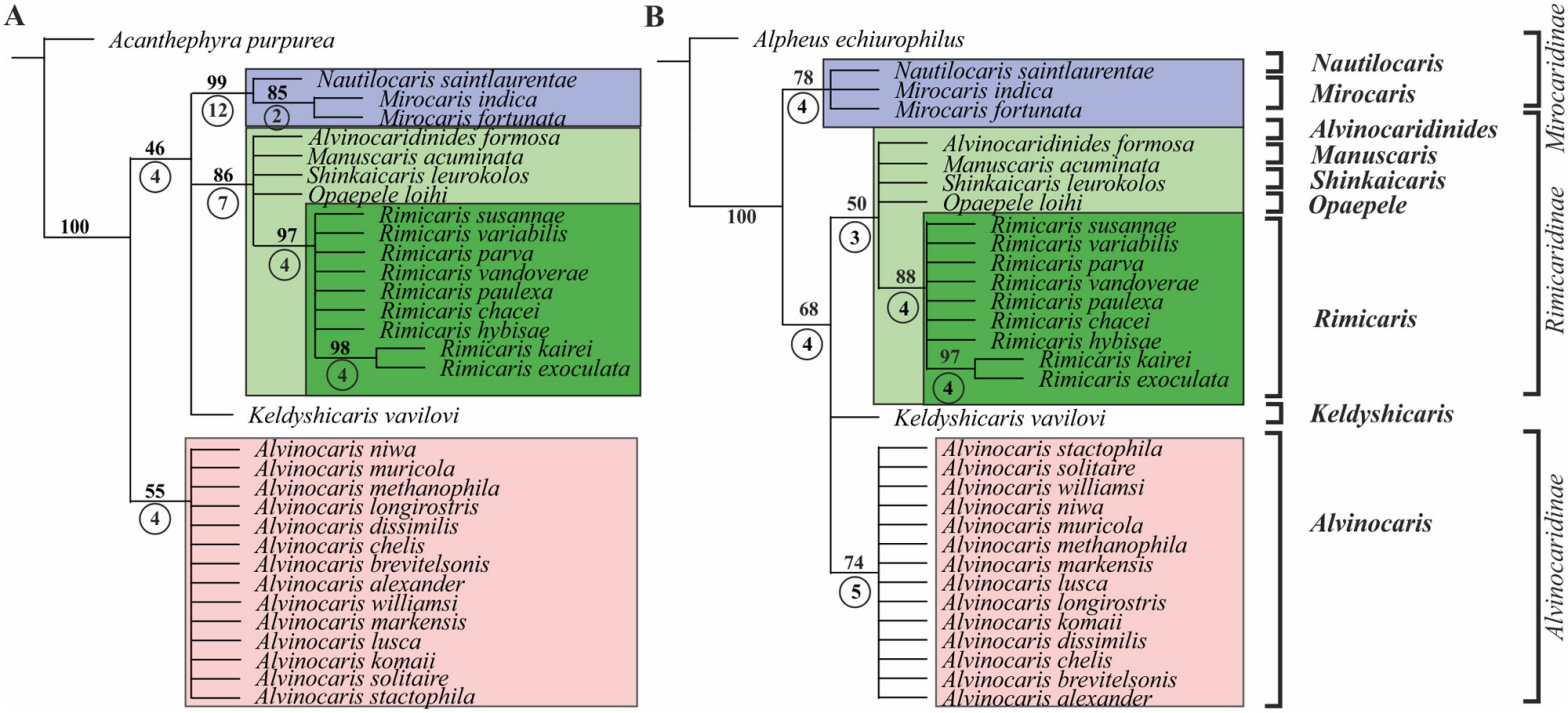
Statistically robust clades of Alvinocarididae with bootstrap support (numbers above the clade) and Bremer support (numbers below the clade in circles). A, analysis 1 with *Acanthephyra purpurea* as outgroup. B, analysis 2 with *Alpheus echiurophilus* as outgroup. Supported clades are marked by different colors: Mirocaridinae (blue), Alvinocaridinae (pink), Rimicaridinae (light green), *Rimicaris* (green).

Analysis 1 with *Acanthephyra purpurea* as the outgroup retrieved 36 minimal length trees of length 145 (Fig 4A). The basal clade *Alvinocaris* (pink in Fig 4A) forms a sister clade to the rest of the family and is followed by *Keldyshicaris* and two sister clades: *Nautilocaris+ Mirocaris* (blue) and *Opaepele+Alvinocaridinides+Manuscaris+Shinkaicaris+Rimicaris* (light green). Within the clade *Alvinocaris*, *Alvinocaris niwa* is the most basal, followed by the other species of *Alvinocaris*. Within the light green clade, there are three clades: *Opaepele*, *Alvinocaridinides+Manuscaris+Shinkaicaris*, and *Rimicaris* (green). After discard of all unsupported clades with Bremer support <3, all considered coloured clades persist (Fig 5A).

Analysis 2 with *Alpheus echiurophilus* as the outgroup retrieved 437 minimal length trees of length 146; the tree topology slightly differs from that in Analysis 1, but the principal clades are the same (Fig 4B, same colours). The clade *Nautilocaris+ Mirocaris* is basal and followed by three clades: (1) *Opaepele+Alvinocaridinides+Manuscaris+Shinkaicaris+Rimicaris*, (2) *Keldyshicaris*, and (3) *Alvinocaris*. After discard of all unsupported clades with Bremer support <3, all considered coloured clades persist (Fig 5B).

### Molecular clades

**Fig 6:**
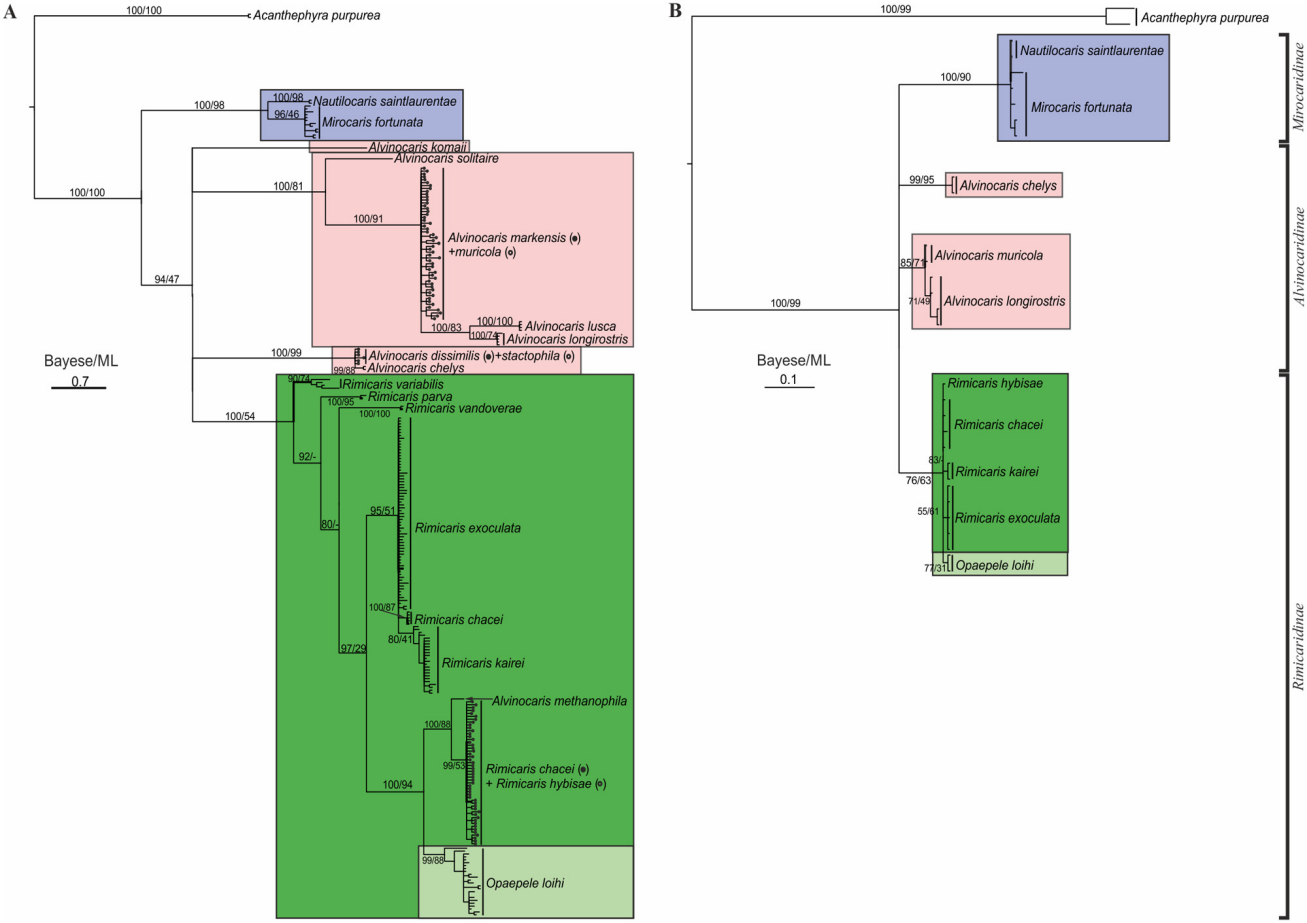
Bayesian phylogenetic trees of the family Alvinocarididae based on COI gene (A) and 16 S gene (B) sequences. The horizontal scale bar marks the number of expected substitutions per site. Statistical support indicated as Bayesian posterior probabilities (left values) and bootstrap analysis with 1,000 replicates (right values) and. Supported clades are marked by different colors: Mirocaridinae (blue), Alvinocaridinae (pink), Rimicaridinae (light green), *Rimicaris* (green).

The molecular phylogenetic Analysis 1 with use of CO1 gene resulted in a tree (Fig 6A), which resembles the morphological tree except the branching of *Alvinocaris* (Fig 5B). In the morpho analysis *Alvinocaris* forms a single clade (not internally resolved), but in the genetic analysis three (COI) or two (16S) clades occur.

***The clade Mirocaris+Nautilocaris*** (Fig 6A, coloured in blue) is supported both by ML bootstrap (98) and Bayesian posterior probability (100). This clade comprises two genera: *Mirocaris* (*M*. *fortunata)* and *Nautilocaris* (*N*. *saintlaurentae)*. Genetic difference between the genera reaches 5.1%. This clade is identical to the clade *Nautilocaris+ Mirocaris* revealed by both morphological analyses. The basal clade is followed by four clades.

**The clade *Alvinocaris komaii*** (Fig 6A, pink) comprises only one species of *Alvinocaris*. Position of this on the tree topology is unresolved and thus requires further research.

**The clade *Alvinocaris solitaire+Alvinocaris markensis+Alvinocaris muricola+ Alvinocaris lusca+Alvinocaris longirostris*** (Fig 6A, pink) is supported by ML bootstrap (81) and Bayesian posterior probability (100). This clade comprises a significant part of the genus *Alvinocaris* (Fig 6A, coloured in pink). *Alvinocaris solitaire* is basal and followed *by A*. *markensis*, *A*. *muricola* and *Alvinocaris lusca+Alvinocaris longirostris*. Genetic differences between three terminal sub-clades range from 5 to 6.7%.

**The clade *Alvinocaris dissimilis +Alvinocaris stactophila +Alvinocaris chelis*** (Fig 6A, pink) is supported by ML bootstrap (99) and Bayesian posterior probability (100). This clade comprises the rest of *Alvinocaris* used in Analysis 1 (Fig 6A, coloured in pink). The former two species are genetically identical, while the genetic distance (p-distance) between them and *A*. *chelys* reaches 0.5%.

**The clade *Rimicaris variabilis + Rimicaris parva + Rimicaris vandoverae + Rimicaris exoculata + Rimicaris chacei* + *Rimicaris kairei* + *Alvinocaris methanophila* + *Rimicaris hybisae+ Opaepele loihi*** (Fig 6A, light green) is supported by Bayesian posterior probability (100). This clade comprises a part of the genus *Rimicaris* (Fig 6A, coloured in green) and two other genera: *Alvinocaris* (a single sequence) and *Opaepele*. Specimens of *C*. *chacei* are divided into two distant groups with genetic differences 7.9%.

The molecular phylogenetic Analysis 2 with use of 16S gene resulted in a tree with similar clades (Fig 6B). The monophyly of the clades ***Mirocaris+Nautilocaris*** and ***Rimicaris exoculata + Rimicaris chacei* + *Rimicaris kairei* + *Rimicaris hybisae+*** was supported (Bayesian posterior probability (100 and 76, respectively). ***Alvinocaris*** created two clades ***Alvinocaris longirostris***+***Alvinocaris muricola*** and ***Alvinocaris chelis*** (Bayesian posterior probability 85 and 99, respectively), which agree with two clades of Analysis 1

## Discussion

Both morphological analyses revealed three major robust monophyletic clades, herein assign to them subfamiliar status, as Mirocaridinae, Rimicaridinae subfam. nov. and Alvinocaridinae subfam. nov.

### The clades

**The clade/subfamily *Mirocaridinae*** (Fig 5A and 5B, blue) comprises three species and two genera: *Nautilocaris* and *Mirocaris*. This clade is supported by the following synapomorphies common for both morpho analyses (starting from the character number):

(48–49) presence of strap-like epipods terminating in a hook, on the fourth pereopod,

(58–60) much reduced appendix interna in second to fourth pleopods

The clade is well supported by molecular data (Fig 6A).

**The clade/subfamily *Rimicaridinae*** (Fig 5A and 5B, light green) comprises thirteen species and five genera: *Opaepele*, *Alvinocaridinides*, *Manuscaris*, *Shinkaicaris*, and *Rimicaris*. This clade is also supported by the following synapomorphies common for both morpho analyses:

(28) entirely fused eyestalks without mould seam,

(47, 54, 57) presence of two or more rows of accessory spinules on the dactyls of the third to fifth pereopods.

The clade is supported by molecular data (Fig 6A). The position of *A*. *methanophila* within Rimicaridinae is worthy of comment. This result is based on a single specimen of *A*. *methanophila*, which was collected at the Blake Ridge Diapir site, sequenced and deposited in GenBank (Accession No AY163260) under the name «*Alvinocaris* sp. TMS-2002». The shrimp was originally named «Blake Ridge shrimp» [43]. Later this specimen along with 33 other adult specimens was described as a new species *A*. *methanophila* [44]. Texeira et al [45] used the same sequence and included this in their clade “ESU 2” which comprised specimens from three genera. We feel that the resulting position of *A*. *methanophila* in a common clade with *R*. *chacei* and *R*. *hybisae* is a result of incorrect identification or processing the material.

Molecular analyses indicate close relation between *Opaepele (O*. *loihi)* and *Rimicaris*, however morphologically they are quite distinct and for now we keep both genera as valid. Both morphological analyses supported validity of remaining genera of the clade Rimicaridinae: *Alvinocaridinides*, *Shinkaicaris*, and *Manuscaris*.

**The clade *Rimicaris*** is nested within the clade Rimicaridinae and comprises all species of the currently recognised genera *Chorocaris* and *Rimicaris* (Fig 5A and 5B, green). This clade received very high support during morphological and molecular analyses and is also supported by the synapomorphies common for both morpho analyses:

(19) dorsal organ under carapace extended beyond the postorbital region,

(61–62) presence of two movable spines mesial to posterolateral tooth on uropodal exopod.

As this is a robust monophyletic clade (except terminal *R*. *exoculata*+*R*. *kairei*), we herein synonymize *Chorocaris* with *Rimicaris*, with Rimicaris being the older name. We note that the type species of *Chorocaris*, *Chorocaris chacei*, was initially described as *Rimicaris* [9]. Our analyses also show that the former *Opaepele susannae* was correctly removed from the genus *Opaepele* (and transferred to *Chorocaris*) [21].

Recent molecular studies by Texeira et al [45] based on studies from the Tropical Atlantic have previously showed a common clade for *Rimicaris* and *Chorocaris*. “These showed very low genetic divergence at levels similar to divergence between individuals of the same species. We posit that these taxa belong to the same genus, possibly even the same species” [45].

The clade is well supported by our molecular data (Fig 6A). A chequerwise arrangement of the species belonging to the former *Rimicaris* and *Chorocaris* within Rimicaridinae gives additional evidence for their genetic similarity and thus synonimising both genera.

One of the unexpected results of our molecular analyses is the position of specimens of *R*. *chacei* in two different and well supported clades (distance 7.9%). Those specimens which are closer to *R*. *exoculata*, were identified and sequenced by T.M. Shank (NN AF125395-397, AF125414-415 from Snake Pit and TAG [17]. The specimens closer to *Opaepele loihi* (NN KC840928-KC840940 from Logatchev and Lucky Strike) are from Teixeira et al. [45]. This division of *R*. *chacei* into two groups may be caused by various factors:

- mitochondrial introgression in which mitochondrial DNA of *R*. *exoculata* may have been incorporated in the populations of *C*. *chacei* at Snake Pit and TAG. Similar effects were found in other marine Malacostraca [46], [47] and also in vent Bivalvia [48], [49],
- existence of cryptic species of *C*. *chacei* recognized as a single morphological species.

**The clade/subfamily *Alvinocaridinae*** (Fig 5A and 5B, pink) comprises fourteen species of the genus *Alvinocaris*. This clade is also supported by the following synapomorphies common for both morpho analyses:

(3, 9) laterally compressed and ventrally carinate rostrum,

(15) presence of postrostral dorsal carina extending beyond the midlength of carapace.

Molecular analyses indicate presence of three supported clades of *Alvinocaris* (Fig 6A). Here we consider the genus and the subfamily as monophyletic on the basis of the two morphological analyses and leave the possibility of a polyphyletic origin of the clade to be resolved by more data in the future.

**The clade *Keldyshicaris*** comprises a single species, *Keldyshicaris vavilovi*. Both morphological analyses did not support monophyly of the former genus *Opaepele* and we suggest division of this genus into two monotypic genera, *Opaepele* with *O*. *loihi* (subfamily Rimicaridinae) and *Keldyshicaris* gen.n. with *K*. *vavilovi* n. comb. The status of this genus may be further clarified after receiving molecular data.

### Morphological trends in Alvinocarididae

Mirocaridinae and Rimicaridinae share a degenerate rostrum, reduced external spines and the presence of the dorsal organ. This type of rostrum may be advantageous in the vicinity of shimmering waters and vent fluids where Mirocaridinae and Rimicaridinae occur. Predators are rare in these extreme biotopes where sulphides, heavy metals, and methane are actively leaching from surrounding rocks [50]. A shorter unarmed rostrum along with reduced spines reduces impact of strong turbulent water fluxes, which are common in the microbiotopes where the shrimps thrive [51].

The dorsal organ has been described inside the carapaces of all recent genera of Mirocaridinae and Rimicaridinae [2], [20], [52–53]. These spot-like organs are believed to be homologous to the ‘dorsal eye’ found in *R*. *exoculata* [2], [54–55], but are smaller and do not comprise four lobes. The dorsal organ, also called ‘dorsal eye’, is an extremely efficient photoreceptor, used for detecting light emitted from the vents [56–58].

Mirocaridinae further differ from other Alvinocarididae in having strap-like epipods terminating in a hook and in much reduced appendix interna in second to fourth pleopods. The strap-like pereopodal epipods are common for many caridean families and these characters may be retained from the ancestor. Modification in pleopodal characters may be related to specific traits in movement or copulation, which may be adaptive in the shimmering waters where Mirocaridinae thrive.

Rimicaridinae possess entirely fused eyestalks and presence of two or more rows of accessory spinules on the dactyls of the third to fifth pereopods—adaptations favouring anchoring close to the strong currents hydrothermal fluids. Within the clade Rimicaridinae, the genus *Rimicaris* shows further modification of external structures on the rostrum and frontal part of carapace, further development of the dorsal organ, and elaboration of uropodal exopod. The polarization of the characters 19 along with inflation of carapace and extension of scaphognathite shows deeper association of *Rimicaris* with vent fluids than any of other genera of the subfamily [51],[59], [60]. Presence of two strong movable spines instead of one (the polarization of characters 61–62) may indicate importance of the tail fan, which is used in the escape behaviour of shrimps ([61]. The presence of additional spines may make this behaviour more efficient when high-temperature turbulent water fluxes can suddenly erupt from the rocks and damage shrimps [51].

Alvinocaridinae are characterized by a well-developed instead of reduced rostrum and postrostral carina, by a frontal ocular tubercle, and by the spination of the fourth-fifth pereopods. These traits are plausibly accounted for by their habitat at the periphery of hydrothermal vent fields [60]. In these habitats, predatory fish do occur and frontal armature may partly protect the shrimps from attacks. The frontal ocular tubercle indicates that the eyes may have additional chemo- or mechanosensory function facilitating orientation at the vent field by means of the frontal tubercle. If so, the tubercle may represent a sensory mechanism alternative to the photoreceptory dorsal organ of other Alvinocarididae. Distal movable spines on the merus of third and fourth pereopods (characters 45 and 51) are present in Alvinocaridinae, potentially enabling more efficient prey catching and sorting and processing the organic particles which the species live on.

## Classification of Alvinocarididae

### Subfamily Mirocaridinae, subfam. nov

urn:lsid:zoobank.org:act:1383E6D1-E57C-4EA0-8CD2-F0CDE9767A97

#### Diagnosis

Carapace dorsally smooth, without postrostral carina; dorsal organ conspicuous; telson bearing 12–19 strong spines. Eyes partly fused, anterior margin of cornea without developed tubercle; epipods of first to fourth pereopods strap-like, terminating in a hook; meri of third and fourth pereopods without movable spines; appendix interna in pleopods II-V much reduced.

#### Type genus

*Mirocaris* Vereshchaka, 1997 [12], by original designation.

#### Genera included

*Mirocaris* Vereshchaka, 1997 [12], *Nautilocaris* Komai, Segonzac, 2004 [14].

#### Remarks

The type species of the genus *Mirocaris* is *M*. *keldyshi*, a junior subjective synonym of *Chorocaris fortunata*. Although the generic status of *Mirocaris* has largely been supported, the family Mirocarididae Vereshchaka, 1997 has not been recognized, but is herein resurrected at subfamily level [19]. Molecular data have been previously indicating high status of *Mirocaris* [17].

### Genus *Mirocaris* Vereshchaka, 1997 [12]

#### Diagnosis

Rostrum dorsoventrally compressed, not reaching end of first antennular segment, apically obtuse, dorsally and ventrally not carinate, smooth; carapace with antennal angle acute, pterygostomial tooth present; dorsal organ restricted to postorbital region; third abdominal segment not serrated; telson with sinuous row of movable dorsolateral spines. Anterior margin of cornea without tubercle; scaphognathite not much expanded, without heavily plumose bacteriophore setae; third maxilliped with distal movable spine on antepenultimate segment; first pereopod with grooming apparatus; second pereopod with a distal movable spine on ischium; ischia of third to fifth pereopods with or without movable spines; dactyli of third to fifth pereopods with a single row of accessory spinules; uropodal exopod with a single movable spine mesial to posterolateral tooth.

#### Type species

*Mirocaris keldyshi Vereshchaka*, *1997 (junior subjective synonym of Chorocaris fortunata* Martin & Christiansen, 1995) [62].

#### Species included

*Mirocaris fortunata* (Martin, Christiansen, 1995) [62], *Mirocaris indica* Komai, Martin, Zala, Tsuchida, Hashimoto, 2006 [63].

#### Remarks

The genus includes 2 species, one from the Atlantic, and the other from the Indian Ocean. Both species are so similar in morphology that supporting molecular data are necessary to prove the validity of *M*. *indica*. We keep here, however, a conservative approach and recognize both species.

### Genus *Nautilocaris* Komai, Segonzac, 2004[14]

#### Diagnosis

Rostrum dorsoventrally compressed, overreaching end of first antennular segment, apically acute, dorsally not carinate, toothed, ventrally smooth; carapace with antennal angle acute, pterygostomial tooth present; dorsal organ restricted to postorbital region; third abdominal segment serrated; telson with sinuous row of movable dorsolateral spines. Anterior margin of cornea without tubercle; scaphognathite not much expanded, without heavily plumose bacteriophore setae; third maxilliped with a distal movable spine on antepenultimate segment; first pereopod with grooming apparatus; second pereopod with distal movable spine on ischium; ischia of third to fifth pereopods with or without movable spines; dactyli of third to fifth pereopods with a single row of accessory spinules; uropodal exopod with a single movable spine mesial to posterolateral tooth.

#### Type species

*Nautilocaris saintlaurentae* Komai & Segonzac, 2004 [14], by monotypy.

#### Species included

*Nautilocaris saintlaurentae* Komai & Segonzac, 2004 [14].

#### Remarks

*Nautilocaris* differs from the closely related genus *Mirocaris* in having a longer and denticulate rostrum and in the serrated pleura of the third abdominal somite.

### Subfamily Rimicaridinae, subfam.nov

urn:lsid:zoobank.org:act:1E84ACE4-B031-43BA-8B91-CD0AFB4DBF77

#### Diagnosis

Carapace dorsally smooth, without postrostral carina; dorsal organ conspicuous; telson bearing 12–19 strong spines. Eyes fused entirely, anterior margin of cornea without developed tubercle; epipods of first to fourth pereopods rudimentary; meri of third and fourth pereopods without movable spines; appendix interna in pleopods II-V developed.

#### Type genus

*Rimicaris* Williams & Rona, 1986 [9], by present designation.

#### Genera included

*Alvinocaridinides* Komai & Chan, 2010 [2], *Manuscaris* Komai & Tsuchida, 2015 [21], *Opaepele* Williams & Dobbs, 1995 [11], *Rimicaris* Williams & Rona, 1986 [9], and *Shinkaicaris* Komai & Segonzac, 2005 [13].

### Genus *Alvinocaridinides* Komai & Chan, 2010 [2]

#### Diagnosis

Rostrum dorsoventrally compressed, not reaching end of first antennular segment, apically blunt, dorsally toothed, ventrally smooth; carapace dorsally smooth, antennal angle acute, pterygostomial tooth present; dorsal organ restricted to postorbital region; third abdominal segment not serrated; telson with sinuous row of movable dorsolateral spines, posterior margin bearing 4 strong spines. Anterior margin of cornea without tubercle; scaphognathite not much expanded, without heavily plumose bacteriophore setae; third maxilliped with 1 distal movable spine on antepenultimate segment; epipods of first to fourth pereopods rudimentary; first pereopod with grooming apparatus; second pereopod without distal movable spine on ischium; ischia of third to fifth pereopods with or without movable spines; dactyli of third to fifth pereopods with two or more rows of accessory spinules; appendix interna in pleopods II-V developed; uropodal exopod with a single movable spine mesial to posterolateral tooth.

#### Type species

*Alvinocaridinides formosa* Komai & Chan, 2010 [2], by original designation and monotypy.

#### Species included

*Alvinocaridinides formosa* Komai & Chan, 2010.

#### Remarks

Komai and Chan [2] established this genus for a single new species, *A*. *formosa*. Our analyses support the validity of this genus.

### *Manuscaris* Komai & Tsuchida, 2015 [21]

#### Diagnosis

Rostrum laterally compressed, reaching end of first antennular segment, apically acute, dorsally carinate, toothed, ventrally smooth; carapace dorsally toothed, pterygostomial tooth present; third abdominal segment serrated; telson with linear row of movable dorsolateral spines, posterior margin bearing 2–4 strong spines. Anterior margin of cornea without tubercle; scaphognathite not much expanded, without heavily plumose bacteriophore setae; third maxilliped with a distal movable spine on antepenultimate segment; epipods of first to fourth pereopods rudimentary; first pereopod with grooming apparatus; second pereopod with distal movable spine on ischium; ischia of third to fifth pereopods with or without movable spines; dactyli of third to fifth pereopods with two or more rows of accessory spinules; appendix interna in pleopods II-V developed; uropodal exopod with a single movable spine mesial to posterolateral tooth.

#### Type species

*Manuscaris acuminata* Komai and Tsuchida, 2015, by monotypy.

#### Species included

*Manuscaris acuminata* Komai and Tsuchida, 2015 [21].

#### Remarks

This genus was recently erected [21] and our analyses support its validity.

### *Opaepele* Williams and Dobbs, 1995 [11]

#### Diagnosis

Rostrum dorsoventrally compressed, not reaching end of first antennular segment, apically blunt, dorsally and ventrally not carinate, notched or smooth; carapace dorsally smooth, antennal angle acute, pterygostomial tooth present; dorsal organ restricted to postorbital region; third abdominal segment serrated; telson with sinuous row of movable dorsolateral spines, posterior margin bearing 2–4 strong spines. Anterior margin of cornea without tubercle; scaphognathite not much expanded, without heavily plumose bacteriophore setae; third maxilliped with a distal movable spine on antepenultimate segment; epipods of first to fourth pereopods rudimentary; first pereopod with grooming apparatus; second pereopod without distal movable spine on ischium; ischia of third to fifth pereopods without movable spines; dactyli of third to fifth pereopods with two or more rows of accessory spinules; appendix interna in pleopods II-V developed; uropodal exopod with a single movable spine mesial to posterolateral tooth.

#### Type species

*Opaepele loihi* Williams, Dobbs, by monotypy.

#### Species included

*Opaepele loihi* Williams, Dobbs, 1995 [11]

#### Remarks

*Opaepele* is herein restricted to the type species only, *O*. *loihi*. *Opaepele susannae* has been recently transferred to *Chorocaris* [21] and our analyses confirm this decision. *Opaepele vavilovi* is transferred to a new genus, *Keldyshicaris* (see below).

### Genus *Rimicaris* Williams & Rona, 1986 [9]

#### Diagnosis

Rostrum if present dorsoventrally compressed, not reaching end of first antennular segment, apically obtuse, dorsally and ventrally not carinate, smooth; carapace dorsally smooth, antennal angle blunt or acute, pterygostomial tooth present or absent; dorsal organ extending beyond the postorbital region; third abdominal segment smooth or serrated; telson with sinuous row of movable dorsolateral spines, posterior margin bearing 2–4 strong spines. Eyes fused entirely, anterior margin of cornea without developed tubercle; scaphognathite expanded, with or without heavily plumose bacteriophore setae; third maxilliped with 0–2 distal movable spines on antepenultimate segment; epipods of first to fourth pereopods rudimentary; first pereopod with or without grooming apparatus; second pereopod without distal movable spine on ischium; ischia of third to fifth pereopods with or without movable spines; dactyli of third to fifth pereopods with two or more rows of accessory spinules; appendix interna in pleopods II-V developed; uropodal exopod with two movable spines mesial to posterolateral tooth.

#### Type species

*Rimicaris exoculata* Williams & Rona, 1986 [9], by original designation.

#### Species included

*Rimicaris chacei* (Williams, Rona, 1986) [9], *Rimicaris exoculata* Williams, Rona, 1986, *Rimicaris hybisae* Nye, Copley, Plouviez, 2012 [3], *Rimicaris kairei* Watabe, Hashimoto, 2002 [64], *Rimicaris parva* (Komai, Tsuchida) [21], *Rimicaris paulexa* (Martin, Shank, 2005) [65], *Rimicaris susannae* (Komai, Giere, Segonzac, 2007) [66], *Rimicaris vandoverae* (Martin, Hessler, 1990) [10], and *Rimicaris variabilis* (Komai, Tsuchida) [21].

#### Remarks

The genus includes all species of the former genera *Chorocaris* sensu Komai and Tsuchida [21] and *Rimicaris*.

*Rimicaris exoculata* from the Atlantic and *R*. *kairei* from the Indian Ocean are very similar in morphology and are not statistically distinct on the molecular tree (Fig 6A). We keep a conservative approach and recognize both species, while pointing out a need of additional research to confirm their validity.

*Rimicaris vandoverae* and *R*. *paulexa* are so similar morphologically that it is impossible to articulate sharp distinctions between them. The species are geographically isolated and the minor morphological distinctions may refer to an inter-population difference rather than to an inter-specific variability. Molecular data (now missing for *R*. *paulexa*) will help in understanding the status of these species.

As stated above, *R*. *chacei* may include at least two cryptic species. New sequences are desirable to clarify this situation.

### Genus *Shinkaicaris* Komai & Segonzac, 2005 [13]

#### Diagnosis

Rostrum laterally compressed, overreaching end of first antennular segment, apically acute, dorsally carinate, toothed, ventrally smooth; carapace dorsally toothed, antennal angle acute, pterygostomial tooth present; dorsal organ restricted to postorbital region; third abdominal segment not serrated; telson with sinuous row of movable dorsolateral spines, posterior margin bearing 2–4 strong spines. Anterior margin of cornea without tubercle; scaphognathite not much expanded, without heavily plumose bacteriophore setae; third maxilliped with a distal movable spine on antepenultimate segment; epipods of first to fourth pereopods rudimentary; first pereopod with grooming apparatus; second pereopod without distal movable spine on ischium; ischia of third to fifth pereopods without movable spines; dactyli of third to fifth pereopods with two or more rows of accessory spinules; appendix interna in pleopods II-V developed; uropodal exopod with a single movable spine mesial to posterolateral tooth.

#### Type species

*Alvinocaris leurokolos* Kikuchi & Hashimoto, 2000 [67], by monotypy.

#### Genera included

*Shinkaicaris leurokolos* Kikuchi, Hashimoto, 2000.

#### Remarks

Our analyses support the validity of the genus.

### Subfamily Alvinocaridinae, subfam. nov

urn:lsid:zoobank.org:act:87404656-6EDE-490E-A8E5-0F2464B370A7

#### Diagnosis

Rostrum laterally compressed, overreaching end of first antennular segment, apically acute, dorsally carinate and toothed, ventrally carinate, toothed or smooth; carapace dorsally toothed or smooth, with postrostral carina extending beyond midlength of carapace, antennal angle acute, pterygostomial tooth present; dorsal organ inconspicuous; third abdominal segment smooth or serrated; telson with linear row of movable dorsolateral spines, posterior margin convex or concave, bearing 4–18 strong spines. Eye partly fused, anterior margin of cornea with developed tubercle; scaphognathite not much expanded, without heavily plumose bacteriophore setae; third maxilliped with 1–2 distal movable spines on antepenultimate segment; epipods of first to fourth pereopods rudimentary; first pereopod with grooming apparatus; second pereopod with distal movable spines on ischium; meri of third and fourth pereopods with movable spines; ischia of third to fifth pereopods with movable spines; dactyli of third to fifth pereopods with a single row of accessory spinules; appendix interna in pleopods II-V developed; uropodal exopod with a single movable spine mesial to posterolateral tooth.

#### Type genus

*Alvinocaris* Williams, Chace, 1982 [8], by present designation

#### Genera included

*Alvinocaris* Williams, Chace, 1982 [8].

### Genus *Alvinocaris* Williams, Chace, 1982 [8]

#### Diagnosis

As in subfamily.

#### Type species

*Alvinocaris lusca* Williams, Chace, 1982 [8], by monotypy.

#### Species included

*Alvinocaris alexander* Ahyong, 2009 [38], *A*. *brevitelsonis* Kikuchi, Hashimoto, 2000 [67], *A*. *chelys* Komai, Chan, 2010 [2], *A*. *dissimilis* Komai, Segonzac, 2005 [13], *A*. *komaii* Zelnio, Hourdez, 2009 [18], *A*. *longirostris* Kikuchi, Ohta, 1995 [7], *A*. *lusca* Williams, Chace, 1982 [8], *A*. *markensis* Williams, 1988 [68], *A*. *methanophila* Komai, Shank, Van Dover, 2005 [69], *A*. *muricola* Williams, 1988 [68], *A*. *niwa* Webber, 2004 [70], *A*. *solitaire* Yahagi, Watanabe, Kojima, Beedessee, Komai, 2014 [71], *A*. *stactophila* Williams, 1988 [68], *A*. *williamsi* Shank, Martin, 2003 [70].

#### Remarks

Molecular data indicate a presence of three species groups at least: (1) *A*. *komaii*, (2) *A*. *solitaire*, *A*. *markensis*, *A*. *muricola*, *A*. *lusca*, *A*. *longirostris*, and (3) A. *dissimilis*, *A*. *stactophila*, *Alvinocaris chelis*. Morphological analyses do not provide robust clades within *Alvinocaris*.

### *Keldyshicaris* gen.nov

urn:lsid:zoobank.org:act:4D7D81BC-2C9A-45A0-93EC-D276E33FA174

#### Diagnosis

Rostrum dorsoventrally compressed, not reaching end of first antennular segment, apically blunt, dorsally not carinate, dorsally and ventrally notched; carapace dorsally smooth, antennal angle acute, pterygostomial tooth present; dorsal organ restricted to postorbital region; third abdominal segment serrated; telson with linear row of movable dorsolateral spines, posterior margin bearing 2–4 strong spines. Anterior margin of cornea with rudimentary tubercle; scaphognathite not much expanded, without heavily plumose bacteriophore setae; third maxilliped with a distal movable spine on antepenultimate segment; epipods of first to fourth pereopods rudimentary; first pereopod with grooming apparatus; second pereopod with distal movable spine on ischium; dactyli of third to fifth pereopods with two or more rows of accessory spinules; appendix interna in pleopods II-V developed; uropodal exopod with a single movable spine mesial to posterolateral tooth.

#### Type species

*Opaepele vavilovi* Lunina and Vereshchaka, 2010.

#### Species included

*Keldyshicaris vavilovi* (Lunina and Vereshchaka, 2010) [20].

#### Etymology

Named after the Russian R/V "Akademik Mstislav Keldysh" which significantly contributed to the studies of vent fauna.

#### Remarks

The proper position and status of *Keldyshicaris* within Alvinocarididae remains uncertain.

### Key to subfamilies, genera, and species of Alvinocarididae (Table 5)

**Table 5:** 

1. Carapace with conspicuous postrostral carina extending beyond the midlength; no conspicuous dorsal organ. Meri of pereopods III-IV with strong movable spines	2 (**subfamily Alvinocaridinae, *Alvinocaris***)
- Carapace without conspicuous postrostral carina extending beyond the midlength; dorsal organ conspicuous; Meri of pereopods III-IV without strong movable spines	13
2. Dorsal teeth present only on rostrum	*Alvinocaris niwa*
- Dorsal teeth both on rostrum and on carapace	3
3. Telson with 2–4 strong spines on posterior margin	4
- Telson with 6 or more strong spines on posterior margin	8
4. No strong movable spines on ischium of fourth pereopods	*Alvinocaris chelys*
- Ischium of fourth pereopod with strong movable spines	5
5. Rostrum not reaching end of second antennular segment	*A*. *methanophyla* in the Atlantic and *A*. *alexander* in the Pacific Ocean
- Rostrum overreaching end of second antennular segment	6
6. Ventral margin of rostrum with 1–2 small subdistal teeth	*Alvinocaris dissimilis*
- Ventral margin of rostrum with 3 or more teeth	7
7. Posterior margin of telson with >2 pairs of spines	*Alvinocaris brevitelsonis*
- Posterior margin of telson with 2 pairs of spines at lateral corners and 12–14 plumose setae	*A*. *muricola* in the Atlantic and *A*. *longirostris* in the Pacific Ocean.
8. Posterior margin of telson concave	9
- Posterior margin of telson convex	11
9. Pleura of third abdominal somite smooth; posterior margin of telson bilobed; dactyli of third to fifth pereopods with two or more rows of accessory spinules	*Alvinocaris komaii*
- Pleura of third abdominal somite serated; posterior margin of telson slightly concave; dactyli of third to fifth pereopods with a single row of accessory spinules	10
10. Rostrum not reaching end of second antennular segment, with 3 or more ventral teeth	*Alvinocaris markensis*
- Rostrum overreaching end of second antennular segment, with 1–2 ventral teeth	*Alvinocaris solitaire*
11. Rostrum with ventral teeth	12
- No ventral teeth on rostrum	*Alvinocaris williamsi*
12. Rostrum with a single ventral tooth. Carapace with 6–10 dorsal teeth	*Alvinocaris stactophyla*
- Rostrum with 2–6 ventral teeth. Carapace with 1–5 dorsal teeth	*Alvinocaris lusca*
13. Epipods of first to fourth pereopods developed, strap-like, ending in hook; appendices internae of second to fourth pleopods rudimentary	14 (**subfamily Mirocaridinae)**
- Epipods of first to fourth pereopods rudimentary, not strap-like, not ending in hook; appendices internae of second to fourth pleopods normally developed	15 (**subfamily Rimicaridinae**)
14. Rostrum not reaching end of first antennular segment, dorsally smooth. Pleura of third abdominal somite smooth	*Mirocaris (M*. *fortunata* in the Atlantic, *M*. *indica* in the Indian Ocean)
- Rostrum overreaching end of first antennular segment, with dorsal teeth. Pleura of third abdominal somite serrate	*Nautilocaris*, the only species *N*. *saintlaurentae*
15. Rostrum overreaching end of 1^st^ antennular segment, carapace with dorsal teeth	16
- Rostrum not reaching end of 1^st^ antennular segment, carapace without dorsal teeth	17
16. Rostrum and carapace bearing a total of 11 or more dorsal teeth; pleura of third abdominal somite serrate; row of dorsolateral spines on telson linear; ischia of third and fourth pereopods with movable spines	genus *Manuscaris*, the only species *Manuscaris acuminata*
Rostrum and carapace bearing a total of 10 or less dorsal teeth; pleura of third abdominal somite not serrate; row of dorsolateral spines on telson sinuous; ischia of third and fourth pereopods without movable spines	*Shinkaicaris*, the only species *Shinkaicaris leurokolos*
17. Rostrum with dorsal teeth or notches; dorsal organ restricted to postorbital region; uropodal exopod with a single movable spine mesial to posterolateral tooth.	18
- Rostrum without dorsal teeth or notches; dorsal organ extending beyond postorbital region; uropodal exopod with two movable spines mesial to posterolateral tooth	20 (genus *Rimicaris*)
18. Rostrum with acute tip, bearing >10 dorsal teeth, ventrally unarmed; pleura of third abdominal somite not serrate	*Alvinocaridinides*, the only species *Alvinocaridinides formosa*.
- Rostrum with blunt tip, bearing <10 dorsal notches, ventrally armed with 1–2 notches; pleura of third abdominal somite serrate	19
19. Telson with sinuous row of dorsolateral spines and 2–4 spines on posterior margin; cornea without anterior tubercle; ischia of third and fifth pereopods without strong movable spines; dactyli of third to fifth pereopods with two or more rows of accessory spinules	*Opaepele*, the only species *Opaepele loihi*.
- Telson with linear row of dorsolateral spines and >10 spines on posterior margin; cornea with anterior tubercle; ischia of third and fifth pereopods with strong movable spines; dactyli of third to fifth pereopods with a single row of accessory spinules	*Keldyshicaris*, the only species *Keldyshicaris vavilovi*
20. Carapace width not exceeding carapace height in adults; dorsal organ nearly entire; scaphognathite without heavily plumose bacteriophore setae	21
- Carapace width exceeding carapace height in adults; dorsal organ four-lobed; scaphognathite with heavily plumose bacteriophore setae	25
21. Dactyli of third to fifth pereopods with two rows of accessory spinules	22
- Dactyli of third to fifth pereopods with 3–4 rows of accessory spinules	23
22. Pleura of fourth and fifth abdominal somites not serrated	*Chorocaris parva*
- Pleura of fourth and fifth abdominal somites serrated	*Chorocaris susannae*
23. Pterigostomial tooth absent	*Chorocaris chacei*
- Pterigostomial tooth present	24
24. Pleura of fourth and fifth abdominal somites serrated	*Chorocaris variabilis*
- Pleura of fourth and fifth abdominal somites not serrated	*Chorocaris vandoverae* from the Mariana Back Arc Basin and *Chorocaris paulexa* from the East Pacific Rise
25. Rostrum short but conspicuous; dorsal organ with a pore; antepenultimate segment of third maxilliped with 1–2 distal movable spines	*Rimicaris hybisae*
- Rostrum absent; dorsal organ without pores; antepenultimate segment of third maxilliped without distal movable spines	*R*. *exoculata-R*. *kairei* complex *(R*. *exoculata* in the Atlantic, *R*. *kairei* in the Indian Ocean)

## References

[1] Martin J, Haney T (2005) Decapod crustaceans from hydrothermal vents and cold seeps: a review through 2005. Zoological Journal of the Linnean Society 145: 445–522.

[2] Komai T, Chan T-Y (2010) A new genus and two new species of alvinocaridid shrimps (Crustacea:Decapoda: Caridea) from a hydrothermal vent field off northeastern Taiwan. Zootaxa 2372: 15–32.

[3] Nye V, Copley J, Plouviez S (2012) A new species of *Rimicaris* (Crustacea: Decapoda: Caridea: Alvinocarididae) from hydrothermal vent fields on the Mid-Cayman Spreading Centre, Caribbean. J Mar Biol Assoc U.K. 92: 1–16.

[4] Fujikura K, Hashimoto J, Fujiwara Y, Okutani T (1995) Community ecology of the chemosynthetic community at Off Hatsushima site, Sagami Bay, Japan. JMSTC Journal of Deep Sea Research 11: 227–241 [in Japanese with English summary]

[5] Fujikura K, Hashimoto J, Fujiwara Y, Okutani T (1996) Community ecology of the chemosynthetic community at Off Hatsushima site, Sagami Bay, Japan-II: comparisons of faunal similarity. JMSTC Journal of Deep Sea Research 12: 133–153 [in Japanese with English summary]

[6] Watabe H, Miyake H (2000) Decapod fauna of the hydrothermally active and adjacent fields on the Hatoma Knoll, southern Japan. JAMSTEC Journal of Deep Sea Research 17: 29–34 [in Japanese with English summary].

[7] Kikuchi T, Ohta S (1995) Two caridean shrimps of the families Bresiliidae and Hippolytidae from a hydrothermal field on the Iheya Ridge, off the Ryukyu Islands, Japan. Journal of Crustacean Biology 15: 771–785.

[8] Williams A, Chace F Jr (1982) A new caridean shrimp of the family Bresiliidae from thermal vents of the Galapagos Rift. Journal of Crustacean Biology 2: 136–147.

[9] Williams A, Rona P (1986) Two new caridean shrimps (Bresiliidae) from a hydrothermal field on the Mid-Atlantic Ridge. J Crustacean Biol 6: 446–462.

[10] Martin J, Hessler R (1990) *Chorocaris vandoverae*, a new genus and species of hydrothermal vent shrimp (Crustacea, Decapoda, Bresiliidae) from the Western Pacific. Contributions in Science 417: 1–11.

[11] Williams A, Dobbs F (1995) A new genus and species of caridean shrimp (Crustacea, Decapoda, Bresiliidae) from hydrothermal vents on Loihi Seamount, Hawaii. Proc. Entomol. Soc. Wash. 108: 228–237.

[12] Vereshchaka A (1997) A new family for a deep-sea caridean shrimp from North Atlantic hydrothermal vents. Journal of the Marine Biological Association of the United Kingdom 77: 425–438.

[13] Komai T, Segonzac M (2005) A revision of the genus *Alvinocaris* Williams and Chace (Crustacea: Decapoda: Caridea: Alvinocarididae), with descriptions of a new genus and a new species of *Alvinocaris* . Journal of Natural History 39: 1111–1175.

[14] Komai T, Segonzac M (2004) A new genus and species of alvinocaridid shrimp (Crustacea: Decapoda: Caridea) from hydrothermal vents on the North Fiji and Lau Basins, south-western Pacific. Journal of the Marine Biological Association of the United Kingdom 84: 1179–1188.

[15] Vereshchaka A (1996) A new genus and species of caridean shrimp (Crustacea: Decapoda: Alvinocarididae) from North Atlantic hydrothermal vents. Journal of the Marine Biological Association of the United Kingdom 76: 951–961.

[16] Martin J, Signorovitch J, Patel H (1997) A new species of *Rimicaris* (Crustacea: Decapoda: Bresiliidae) from the Snake Pit hydrothermal vent field on the Mid-Atlantic Ridge. Proceedings of the Biological Society of Washington 110: 399–411.

[17] Shank T, Black M, Halanych K, Lutz R, Vrijenhoek R (1999) Miocene radiation of deep-sea hydrothermal vent shrimp (Caridea: Bresiliidae): evidence from mitochondrial cytochrome oxidase subunit I. Molecular Phylogenetics and Evolution 13: 244–54. 10603254 10.1006/mpev.1999.0642

[18] Zelnio K, Hourdes S (2009) A new species of *Alvinocaris* (Crustacea: Decapoda: Caridea: Alvinocarididae) from hydrothermal vents at the Lau Basin, southwest Pacific, and a key to the species of Alvinocarididae. Proc. Entomol. Soc. Wash. 122: 52–71.

[19] De Grave S, Fransen C (2011) Carideorum Catalogus: The Recent Species of the Dendrobranchiate, Stenopodidean, Procarididean and Caridean Shrimps (Crustacea: Decapoda) NCB Naturalis. (pp. 196–304).

[20] Lunina A, Vereshchaka A (2010) A new vent shrimp (Crustacea: Decapoda: Alvinocarididae) from the Mid-Atlantic Ridge. In: De Grave S, Fransen C (eds.), Contributions to shrimp taxonomy. Zootaxa 2372: 69–74.

[21] Komai T, Tsuchida S (2015) New records of Alvinocarididae (Crustacea: Decapoda: Caridea) from the southwestern Pacific hydrothermal vents, with descriptions of one new genus and three new species. Journal of Natural History (in press). 10.1080/00222933.2015.1006702

[22] Tokuda G, Yamada A, Nakano K, Arita N, Yamasaki H (2006) Occurrence and recent long-distance dispersal of deep-sea hydrothermal vent shrimps. Biology letters 2: 257–260. 17148377 10.1098/rsbl.2005.0420 PMC1618913

[23] Bonnivard E, Catrice O, Ravaux J, Brown S, Higuet D (2009) Survey of genome size in 28 hydrothermal vent species covering 10 families. Genome 52: 524–536. 10.1139/g09-027 19483771

[24] Pedersen RB, Rapp HT, Thorseth IH, Lilley MD, Barriga FJ, Baumberger T et al (2010) Discovery of a black smoker vent field and vent fauna at the Arctic Mid-Ocean Ridge. Nat Commun 1: 126 21119639 10.1038/ncomms1124 PMC3060606

[25] Yang J, Lu B, Chen D, Yu Y, Yang F, Nagasawa H. et al (2013). When did decapods invade hydrothermal vents? Clues from the Western Pacific and Indian Oceans. Molecular Biology and Evolution 30: 305–309. 10.1093/molbev/mss224 23002089

[26] Teixeira S, Serrão E, Arnaud-Haond S (2012) Characterization of 15 polymorphic microsatellite loci in *Rimicaris exoculata*, and cross-amplification in other hydrothermal-vent shrimp. Conservation Genetics Resources 4: 81–84.

[27] Vereshchaka A (2000) Revision of the genus *Sergia* (Decapoda: Dendrobranchiata: Sergestidae): Taxonomy and distribution. Galathea Report 18, 69–207.

[28] Vereshchaka A (1997) Comparative morphological studies on four populations of the shrimp *Rimicaris exoculata* from the Mid-Atlantic ridge. Deep-Sea Research I, V. 44 (11), p. 1905–1921.

[29] Lunina A, Vereshchaka A (2008) Hydrothermal vent shrimps *Alvinocaris markensis*: interpopulation variation. Dokl Biol Sci 421: 266–268. 18841811 10.1134/s0012496608040133

[30] Lunina A (2011) Vent shrimps of the Mid-Atlantic Ridge. PhD Thesis, P.P. Shirshov Institute of Oceanology of RAS, Russia, Moscow [in Russian]

[31] Li CP, De Grave S, Chan TY, Lei HC, Chu KH (2011) Molecular systematics of caridean shrimps based on five nuclear genes: implications for superfamily classification. Zoologischer Anzeiger-A Journal of Comparative Zoology 250: 270–279.

[32] Milne-Edwards A (1881) Compte rendu sommaire d’une exploration zoologique faite dand l’Atlantique, à bord du navire le Travailleur. Comptes Rendus hebdomadaires des Séances de l’Académie des Sciences 93: 931–936. 17597171

[33] Wong MV, Pérez-Moreno JL, Chan T-Y, Frank TM, Bracken-Grissom HD (2015) Phylogenetic and transcriptomic analyses reveal the evolution of bioluminescence and light detection in marine deep-sea shrimps of the family Oplophoridae (Crustacea: Decapoda). Mol. Phyl Evol 83: 278–292. 10.1016/j.ympev.2014.11.013 25482362

[34] Anker A, Komai T, Marin IN (2015) A new echiuran-associated snapping shrimp (Crustacea: Decapoda: Alpheidae) from the Indo-West Pacific. Zootaxa 3914: 441–455. 10.11646/zootaxa.3914.4.4 25661953

[35] Nixon K (1999). The parsimony ratchet, a new method for rapid parsimony analysis. Cladistics 15: 407–414. 10.1111/j.1096-0031.1999.tb00277.x 34902938

[36] Goloboff P, Farris S, Nixon K (2000) TNT (Tree analysis using New Technology).

[37] Maddison W, Maddison D (2001) Mesquite: a modular system for evolutionary analysis.

[38] Ahyong S (2009) New Species and New Records of Hydrothermal Vent Shrimps from New Zealand (Caridea: Alvinocarididae, Hippolytidae). Crustaceana 82: 775–794.

[39] Wang L, Jiang T (1994) On the complexity of multiple sequence alignment. J. Comput. Biol. 1: 337–348. 8790475 10.1089/cmb.1994.1.337

[40] Darriba D, Taboada GL, Doallo R, Posada D. (2012) jModelTest 2: more models, new heuristics and parallel computing. Nature Methods 9: 772 10.1038/nmeth.2109 22847109 PMC4594756

[41] Felsenstein J (1985) Confidence limits on phylogenies: an approach using the bootstrap. Evolution 39: 783–791. 28561359 10.1111/j.1558-5646.1985.tb00420.x

[42] Ronquist F, Hulsenbeck J (2003) MrBayes 3: Bayesian phylogenetic inference under mixed models. Bioinformatics 19:1572–1574. 12912839 10.1093/bioinformatics/btg180

[43] Van Dover CL, · Van Dover P, · Aharon JM, · Bernhard E, · Caylor M, · Doerries W · et. al (2003) Blake Ridge methane seeps: characterization of a soft-sediment, chemosynthetically based ecosystem. Deep Sea Research Part I: Oceanographic Research Papers 50: 281–300.

[44] Komai T, Shank TM, Van Dover CL (2005) A new species of *Alvinocaris* (Crustacea: Decapoda: Caridea: Alvinocarididae) and a new record of *A*. *muricola* from methane seeps on the Blake Ridge Diapir, Northwestern Atlantic. Zootaxa, 1019: 27–42.

[45] Teixeira S, Olu K, Decker C, Cunha RL, Fuchs S, Hourdez S. et al (2013) High connectivity across the fragmented chemosynthetic ecosystems of the deep Atlantic Equatorial Belt: efficient dispersal mechanisms or questionable endemism? Molecular Ecology, 22: 4663–4680. 10.1111/mec.12419 23927457

[46] Audzijonyte A, Väinölä R (2007) *Mysis nordenskioldi* n. sp. (Crustacaea, Mysida), a circumpolar coastal mysid separated from the NE Pacific *M*. *litoralis* (Banner, 1948). Polar Biol. 30: 1137–1157.

[47] Darling JA (2011) More than one way to invade: lessons from genetic studies of Carcinus shore crabs In the Wrong Place-Alien Marine Crustaceans: Distribution, Biology and Impacts. Springer Netherlands P. 661–685.

[48] O’Mullan GD, Maas PAY, Lutz RA, Vrijenhoek RC (2001) A hybrid zone between hydrothermal vent mussels (Bivalvia: Mytilidae) from the Mid-Atlantic Ridge. Mol. Ecol. 10: 2819–2831. 11903895 10.1046/j.0962-1083.2001.01401.x

[49] Won Y, Hallam SJ, O’Mullan GD, Vrijenhoek RC (2003) Cytonuclear disequilibrium in a hybrid zone involving deep-sea hydrothermal vent mussels of the genus *Bathymodiolus* . Mol. Ecol. 12: 3185–3190. 14629398 10.1046/j.1365-294x.2003.01974.x

[50] Renninger GH, Kass L, Gleeson RA, VanDover CL, Battelle BA, Jinks RN, Herzog ED, Chamberlain SC (1995) Sulphide as a chemical stimulus for deep-sea hydrothermal vent shrimp. Biological Bulletin 189: 69–76. 27768499 10.2307/1542456

[51] Vereshchaka AL (1997) Comparative morphological studies on four populations of the shrimp *Rimicaris exoculata* from the Mid-Atlantic Ridge. Deep Sea Research Part I: Oceanographic Research Papers 44: 1905–1921.

[52] Desbruye`res D, Segonzac M and Bright M. (2006) Handbook of deep-sea hydrothermal vent fauna. Vienna: Biologiezentrum der Oberosterreichische Landesmuseen.

[53] Tsuchida S., Yamaguchi T., Komai T and Watanabe H. (2008) Arthropoda In Fujikura K., Okutani T. and Maruyama T. (eds) Deep-sea life—biological observations using research submersibles. Hatano: Tokai University Press, pp.100–178.

[54] Kuenzler R.O, Kwasniewski J.T., Jinks R.N., Lakin R.C., Battelle B.-A., Herzog E.D., Renninger G.H. and Chamberlain S.C. (1997) Retinal anatomy of new bresiliid shrimp from the Lucky Strike and Broken Spur hydrothermal vent fields on the Mid-Atlantic Ridge. Journal of the Marine Biological Association of the United Kingdom 77, 707–725.

[55] Lakin R.C., Jinks R.N. Batelle B.-A., Herzog E.D., Kass L., Renninger G.H. and Chamberlain S.C. (1997) Retinal anatomy of *Chorocaris chacei*, a deep-sea hydrothermal vent shrimp from the Mid-Atlantic Ridge. Journal of Comparative Neurology 383, 503–514. 9302103

[56] Pelli DG, Chamberlain SC (1989) The visibility of 350 C black-body radiation by the shrimp *Rimicaris exoculata* and man. Nature 337: 460–461. 15726721 10.1038/337460a0

[57] O'Neill PJ, Jinks RN, Herzog ED, Battelle BA, Kass L, Renninger GH, Chamberlain SC (1995) The morphology of the dorsal eye of the hydrothermal vent shrimp, *Rimicaris exoculata* . Visual neuroscience 12: 861–875. 8924410 10.1017/s0952523800009421

[58] Nye V, Copley J, Plouviez S (2011) A new species of *Rimicaris* (Crustacea:Decapoda: Caridea: Alvinocarididae) from hydrothermal vent fields on the Mid-Cayman Spreading Centre, Caribbean. J Mar Biol Assoc U.K. 92: 1–16.

[59] Gebruk A, Galkin S, Vereshchaka AL, Moskalev LI, Southward A (1997) Ten years of exploration of Atlantic hydrothermal fauna: results and problems. Advances in Marine Biology, 32: 93–144.

[60] Vereshchaka AL, Vinogradov ME (2002) Three-dimensional view of the Atlantic abyssal benthopelagic vent community. Cahiers de Biologie Marine 43: 303–305.

[61] Vereshchaka AL, Olesen J, Lunina AA (2014) Global diversity and phylogeny of pelagic shrimps of the former genera *Sergestes* and *Sergia* (Crustacea, Dendrobranchiata, Sergestidae), with definition of eight new genera. PloS one 9 (11), e112057 10.1371/journal.pone.0112057 25409458 PMC4237343

[62] Martin J, Christiansen J (1995) A new species of the shrimp genus *Chorocaris* Martin, Hessler, 1990 (Crustacea: Decapoda: Bresiliidae) from hydrothermal vent fields along the Mid-Atlantic Ridge. Proceedings of the Biological Society of Washington 108: 220–227.

[63] Komai T, Martin J, Zala K, Tsuchida S, Hashimoto J (2006) A new species of *Mirocaris* (Crustacea: Decapoda: Caridea: Alvinocarididae) associated with hydrothermal vents on the Central Indian Ridge, Indian Ocean. Scientia Marina 70: 109–119.

[64] Watabe H, Hashimoto J (2002) A new species of the genus *Rimicaris* (Alvinocarididae: Caridea: Decapoda) from the active hydrothermal vent field,”Kairei Field”, on the central Indian Ridge, the Indian Ocean. Zoological Science 19: 1167–1174. 12426479 10.2108/zsj.19.1167

[65] Martin J, Shank T (2005) A new species of the shrimp genus *Chorocaris* (Decapoda: Caridea: Alvinocarididae) from hydrothermal vents in the eastern Pacific Ocean. Proceedings of the Biological Society of Washington 118: 183–198.

[66] Komai T, Giere O, Segonzac M (2007) New record of alvinocarid shrimps (Crustacea: Decapoda: Caridea) from hydrothermal vent fields on the southern Mid-Atlantic Ridge, including a new species of the genus *Opaepele* . Species Diversity 12: 237–253.

[67] Kikuchi T, Hashimoto J (2000) Two new caridean shrimps of the family Alvinocarididae (Crustacea, Decapoda) from a hydrothermal vent field at the Minami-Ensei Knoll in the Mid-Okinawa Trough, Japan. Species Diversity 5: 135–148.

[68] Williams A (1988) New marine decapod crustaceans from waters influenced by hydrothermal discharge, brine, and hydrocarbon seepage. Fishery Bulletin 86: 263–287.

[69] Komai T, Shank T, Van Dover CL (2005) A new species of Alvinocaris (Crustacea: Decapoda: Caridea: Alvinocarididae) and a new record of *A*. *muricola* from methane seeps on the Blake Ridge, Diapir, Northwestern Atlantic. Zootaxa 1019: 27–42.

[70] Webber W (2004) A new species of Alvinocaris (Crustacea: Decapoda: Alvinocarididae) and new records of alvinocaridids from hydrothermal vents north of New Zealand. Zootaxa 444: 1–26.

[71] Yahagi T, Watanabe H, Kojima S, Beedessee G, Komai T (2014) First record and a new species of *Alvinocaris* Williams & Chace, 1982 (Crustacea: Decapoda: Caridea: Alvinocarididae) from the Indian Ocean. Zootaxa 3893 (1): 101–113. 10.11646/zootaxa.3893.1.4 25544513

[72] Hiraoka R, Tsuchida S, Komai T (2013) *Alvinocaris dissimilis* mitochondrial COI gene for cytochrome oxidase subunit I, partial cds, isolate: ad1coi. published only in GenBank: http://www.ncbi.nlm.nih.gov/nuccore/AB779491 AB779492 AB779493 AB779494

[73] Tokuda G, Kumara RP, Yamasaki H (2014) published only in GenBank http://www.ncbi.nlm.nih.gov/nuccore/AB821296

[74] Chan TY, Lei HC, Li CP, Chu KH (2010) Phylogenetic analysis using rDNA reveals polyphyly of Oplophoridae (Decapoda: Caridea). Invertebrate Systematics 24: 172–181.

[75] Hourdez SM, Zelnio KA (2008) published only in GenBank. Available: http://www.ncbi.nlm.nih.gov/genbank/

[76] Bracken HD, De Grave S, Felder DL (2009) Phylogeny of the infraorder Caridea based on mitochondrial and nuclear genes (Crustacea: Decapoda). In Decapod crustacean phylogenetics: 274–298.

[77] Yang CH, Tsang LM, Chu KH, Chan TY (2012) Complete mitogenome of the deep-sea hydrothermal vent shrimp *Alvinocaris chelys* Komai and Chan, 2010 (Decapoda: Caridea: Alvinocarididae). Mitochondrial DNA 23: 417–419. 10.3109/19401736.2012.710212 22943309

[78] Leignel V, Van Wormhould A, Bui QT, Ravallec R, Laulier M (2006) published only in GenBank. Available: http://www.ncbi.nlm.nih.gov/nuccore/ AM076958 AM076958 AM076959

[79] Leignel V, Van Wormhould A, Bui QT, Ravallec R, Laulier M (2008) published only in GenBank. Available: http://www.ncbi.nlm.nih.gov/nuccore/ AM087916 AM087917 AM087918 AM087919 AM087920 AM087921 AM087922 AM087923 AM087924 AM087925

[80] Kim SJ, Pak SJ, Ju SJ (2013) Mitochondrial genome of the hydrothermal vent shrimp *Nautilocaris saintlaurentae* (Crustacea: Caridea: Alvinocarididae). Mitochondrial DNA (0): 1–2. 10.3109/19401736.2013.815169 23876191

[81] Jones WJ, Tunnicliffe V, Jupiter SK, Limen H, Webber R, Vrijenhoek RC (2008) published only in GenBank http://www.ncbi.nlm.nih.gov/nuccore/DQ328838.1

[82] Shank TM, Lutz RA, Vrijenhoek RC (1998) Molecular systematics of shrimp (Decapoda: Bresiliidae) from deep-sea hydrothermal vents, I: Enigmatic "small orange" shrimp from the Mid-Atlantic Ridge are juvenile *Rimicaris exoculata* . Mol Mar Biol Biotechnol. 2:88–96. 9628005

[83] Teixeira S, Cambon-Bonavita M-A, Serrão E, Desbruyéres D, Arnaud-Haond S (2011) Recent population expansion and connectivity in the hydrothermal shrimp *Rimicaris exoculata* along the Mid-Atlantic Ridge. Journal of Biogeography, 38: 564–574.

[84] Beedessee G, Watanabe H, Ogura T, Nemoto S, Yahagi T, Satoshi N, et al (2013) High Connectivity of Animal Populations in Deep-Sea Hydrothermal Vent Fields in the Central Indian Ridge Relevant to Its Geological Setting. PLoS ONE 8(12): e81570 10.1371/journal.pone.0081570 24358117 PMC3864839

[85] Plouviez S, Jacobson A, Wu M, Van Dover CL (2015) Characterization of vent fauna at the Mid-Cayman Spreading Center. Deep Sea Research Part I: Oceanographic Research Papers 97: 124–133.

[86] Bucklin A, Bucklin BD, Ortman RM, Jennings LM, Nigro CJ, Sweetman NJ et al (2010) A “Rosetta Stone” for metazoan zooplankton: DNA barcode analysis of species diversity of the Sargasso Sea (Northwest Atlantic Ocean). Deep Sea Research Part II: Topical Studies in Oceanography 57: 2234–2247.

[87] Wong JM, Pérez-Moreno JL, Chan TY, Frank TM, Bracken-Grissom HD (2015) Phylogenetic and transcriptomic analyses reveal the evolution of bioluminescence and light detection in marine deep-sea shrimps of the family Oplophoridae (Crustacea: Decapoda). Molecular phylogenetics and evolution 83: 278–292. 10.1016/j.ympev.2014.11.013 25482362

